# Findings from the Process Evaluation of a Mobile Health Clinic Designed to Improve Equity of Access to Primary Healthcare for People with Substance Use Disorders and/or Homelessness in One Region in the North East of England, UK

**DOI:** 10.3390/healthcare14050670

**Published:** 2026-03-06

**Authors:** Emma-Joy Holland, Eleanor Ash, Elizabeth Titchener, Sarah Schonewald, Amy O’Donnell, Sedighe Hosseini-Jebeli, Emma A. Adams, Sarah Lonbay, Floor Christie-de Jong, Sarah Norman, Katherine Jackson

**Affiliations:** 1 Population Health Sciences Institute, Faculty of Medical Sciences, Newcastle University, Newcastle upon Tyne NE2 4AX, UK; emma.holland@newcastle.ac.uk (E.-J.H.); eleanor.ash@newcastle.ac.uk (E.A.); liz.titchener@newcastle.ac.uk (E.T.); amy.odonnell@newcastle.ac.uk (A.O.); emma.adams@newcastle.ac.uk (E.A.A.); 2 Recovery Connections, Sunderland NE38 7NQ, UK; 3 Health Economic Unit, Department of Applied Health Sciences, University of Birmingham, Birmingham B15 2TT, UK; s.s.hosseinijebeli@bham.ac.uk; 4 School of Social Sciences and Law, Sunderland University, Sunderland SR6 0DD, UK; sarah.lonbay@sunderland.ac.uk; 5 School of Medicine, Sunderland University, Sunderland SR1 3SD, UK; floor.christie-dejong@sunderland.ac.uk; 6 Sunderland City Council, Sunderland SR1 3AA, UK; sarah.norman@sunderland.gov.uk

**Keywords:** substance use, healthcare access, mobile health clinic, multiple and complex needs, RE-AIM, process evaluation, community interventions, relational support, interdisciplinary

## Abstract

**Background/Objectives**: New models of care are needed to address the barriers people who use substances (PWUS) and/or experience homelessness face when accessing primary healthcare. This study reports findings from the evaluation of a six-month pilot of a mobile health clinic (MHC) co-delivered by primary healthcare, local government, and lived-experience recovery organisations in the North East of England, UK. **Methods**: Pragmatic mixed-methods process evaluation with data sources including a patient survey, overt observations, qualitative interviews, and routine patient data. Qualitative data were analysed using inductive and deductive thematic analysis; quantitative data were analysed descriptively. RE-AIM framework dimensions were applied to inform interpretation. **Results**: N = 164 patients accessed the bus between 1 April and 31 October 2025, with survey data indicating that most patients were PWUS (n = 96, 84%), with experience of homelessness (n = 67, 61%) and/or lived in the most deprived neighbourhoods, with complex physical and mental health needs (Reach). Patients expressed satisfaction with the service, valuing the compassionate and comprehensive support provided. There was qualitative evidence of further re-engagement with statutory healthcare following attendance on the bus (Effectiveness). Local organisations were mostly keen to be involved in the pilot, with participation benefiting from existing local relationships and infrastructure (Adoption). The flexible yet consistent approach of those involved in service delivery was viewed as positive. There was some uncertainty around the functions of the bus and the role of some delivery staff (Implementation). Limited funding was perceived as a barrier to sustaining the bus, alongside lack of capacity within local organisations (Maintenance). **Conclusions**: The study highlighted the positive impact that an MHC can have on this marginalised population and provides further evidence for the need for clinical care that provides relational support and attends to the social determinants of health. The study indicates the potential for interdisciplinary working to improve access to healthcare for PWUS, and underlines that delivering healthcare at a neighbourhood level is reliant on strong community networks. Wider system change is still needed to further support the population.

## 1. Introduction

Substance use disorders (SUDs) are patterns of mild, hazardous or dependent alcohol or illicit drug use that cause damage to an individual’s physical and/or mental health and can lead to clinically significant functional impairment or distress [1]. Dependence is broadly characterised by the substance taking priority over other aspects of a person’s life, diminished control over the substance use, and the continuation of harm despite negative effects [1]. SUDs are associated with social and economic harms to individuals and communities, and a range of acute and chronic health conditions, including liver disease, respiratory infections/disease, cardiovascular disease, infectious diseases including HIV, certain cancers and wounds [2,3]. Internationally, SUDs are a significant cause of premature mortality [3]. In 2024, in England, UK, there were 5565 recorded deaths related to drug misuse (93.0 deaths per million) and the majority involved an opioid or an opiate [4]. The latest data from 2023 for England indicates alcohol-related deaths equated to 40.7 per 100,000 population [5].

The harms from SUDs are a persistent cause of global health inequalities, as they affect people living in deprivation and/or experiencing poverty the most [6]. In England specifically, in 2023, the rate of alcohol-specific mortality in the most deprived areas was double the rate in the least deprived areas (20.9 and 9.8 per 100,000 population respectively) [7], and similar disparities in rates of drug-related deaths are observed [8,9]. With a higher concentration of deprived neighbourhoods found in the North of England compared to other parts of the country, this disparity is also reflected in regional drug and alcohol related morbidity and mortality rates. The North East Region has the highest rates of so called ‘deaths of despair’ related to drug poisoning, alcohol and suicide in England [10]. This notion of ‘despair’ reflects the point that deprivation is a causal factor contributing to SUD and that SUD can be a psychosocial coping mechanism for people when experiencing poverty, housing insecurity, and trauma [11]. The preventative care, treatment and ongoing support that people with SUD (from here referred to as PWUS) can access, is also shaped by associated social and economic adversities. For example, it is common that PWUS experience other harms such as homelessness or insecure housing, mental ill health, criminal justice involvement and domestic abuse (for women in particular) [12]. People experiencing a combination of two or more of these harms can be described as experiencing multiple and complex needs [12,13]. This term reflects the recognition that health and social needs can be both a driver and an outcome to substance use, therefore they cannot be addressed separately and instead require integrated, coordinated responses from services that effectively deliver care [12].

The barriers to PWUS accessing traditional primary and secondary healthcare operate at overlapping patient, provider and system levels [14,15]. At an individual level, whilst qualitative research indicates PWUS worry about their health, e.g., ref. [16], ongoing alcohol or drug dependence may mean people lack motivation or confidence to attend healthcare appointments [17]. For people experiencing homelessness, not having a fixed address can further limit their ability to prioritise health when their basic needs like food and shelter are not being met [17,18]. In the United States (US) and other countries where there is an insurance model of healthcare, material finances present a notable barrier to accessing care for PWUS [17,19,20]. Yet in the UK, where healthcare is free at the point of access via the National Health Service (NHS), material poverty still remains a barrier to access, such as the need for patients to pay for transport to general practitioners (GPs) who provide primary healthcare, or secondary healthcare hospitals [21]. Additionally, with an increasing focus on digital care provision in the UK and elsewhere (currently primary healthcare relies on digital online appointment systems), people without access to technology or with low digital literacy are experiencing growing exclusion from healthcare [22,23,24]. Critically, the way healthcare services are currently configured does not appear to recognise issues that might affect the engagement of PWUS and seems widely disconnected from their needs. For example, having multiple and complex needs can often involve people needing to coordinate their own care across different settings e.g., people need to remember various appointments when they have little help from others and few material resources [25].

Stigma (both interactional and structural) is a further key factor internationally recognised as impacting on the quality of care PWUS receive and acting as a barrier to this population accessing healthcare [21,26,27,28]. In different countries and healthcare settings PWUS have reported feeling judged and lacking in value when accessing primary and secondary healthcare [17,29]. These experiences can contribute to feelings of negative self-worth and can also lead people to disengage with healthcare, meaning inequalities continue to widen. Conversely, there is evidence that when people trust their healthcare clinician and feel like they are valued and respected, they are more likely to engage with healthcare, e.g., ref. [30]. Community and voluntary sector services and staff who work in them are often perceived to be good at providing non-judgemental support [31]. However, these types of services are unequally dispersed across geographic areas [25], and there is evidence these organisations can struggle to implement statutory primary and secondary healthcare interventions for various reasons including lack of staff, finances or guidance on how best to adapt an intervention for the target population [32].

New models of statutory healthcare are urgently needed that are better aligned with PWUS needs, acknowledge the compounding impact of poverty and social conditions on substance use, and recognise the importance of compassionate, relational care in supporting people’s autonomy. Such innovations benefit from involving community and voluntary services that often provide more non-judgemental support and address wider social determinants of health and wellbeing beyond simply clinical care [33]. For example, adopting relational support from people with lived experience of substance use (often referred to as ‘peer link workers’ or ‘recovery navigators’) alongside clinical healthcare can provide a holistic approach to health that is better aligned with PWUS needs, through rapport building, providing stigma-free emotional support, and continuing care beyond clinical appointments, e.g., ref. [34,35] Another approach that has been adopted in different international contexts, but particularly in the US, is the mobile health clinic (MHC), in which care providers see patients in a suitably adapted vehicle that serves as a mobile health clinic. There is some evidence, mostly from observational studies from the US, to suggest that MHCs and other outreach approaches may improve health outcomes for people with multiple and complex needs through increased health screenings, preventative care, and chronic disease management as well as reduced emergency department utilisation [36,37,38,39]. Findings also suggest that they are positively received by patients [40]. However, most of the evidence is from US-based studies, highlighting the need for further research to understand how such models could be implemented within a UK context for people with multiple and complex needs to assess their accessibility and potential outcomes for UK populations.

In this paper we report findings from the process evaluation of a six-month pilot of an MHC intervention that was intended to increase equity and quality of care by providing more immediate primary healthcare in a way that was accessible to PWUS, without stigma and with embedded relational peer support. The longer-term intentions were to help facilitate more sustained engagement with traditional primary healthcare services alongside wider support related to the social determinants of health, and to upskill the primary healthcare workforce. We use relevant constructs of Glasgow et al.’s [41] RE-AIM framework (described in materials and methods) to understand whether the intervention could reach the target population, whether it could effectively provide quality care and how it was implemented in practice. We were also interested in the adoption of the intervention and making recommendations for how this or a similar service could be maintained over the long-term:

Our aims were:

1. To describe the demographic profile and the physical health, mental health and social care needs of the cohort of patients who access the intervention (REACH)
2. To understand how the target population are supported by this service (including patient satisfaction), including any referral pathways (EFFECTIVENESS/IMPLEMENTATION)
3. To consider what has worked well about the delivery model during this pilot, as well as identifying any key challenges to care delivery and making recommendations for future delivery (ADOPTION, IMPLEMENTATION and MAINTENANCE).

## 2. Materials and Methods

### 2.1. Setting

The study was undertaken in Sunderland which is a coastal city in the North East of England, UK, inhabited by approximately 274,200 residents (in 2021), of predominantly White ethnicity (94.6%) and an average age of 43 years [42]. It is the 27th most deprived local government region in England (out of 153, with the 1st being the most deprived) and faces significant and worsening socio-economic challenges, with rising rates of poverty and unemployment, and homeless applications [43,44,45]. Substance use is a prevalent health concern for the city; in 2023 Sunderland had one of the highest rates of alcohol-related mortality in England at 63.4 per 100,000, significantly above the English average (40.7) [43].

### 2.2. Intervention—Co-Designed Mobile Health Clinic for People Experiencing Drug and Alcohol Related Harms and or/Experiencing Homelessness

Recognising the need to increase health equity for PWUS in Sunderland, the MHC intervention (hereafter referred to as the ‘bus’) was designed to improve access to primary healthcare for a target population who were people at risk of alcohol or drug use related harms, and/or who may be experiencing homelessness living in the study region (from here we refer to this group as either the ‘target population’ or just ‘PWUS’, although the target population also included people experiencing homelessness). The intervention was developed as part of a co-design project (the Sunderland PLUS study) during a formative stage of the study which involved qualitative interviews, focus groups, and workshops with individuals with lived experience of SUD, carers, and professionals. Key principles were identified which guided intervention development, including the need for a flexible mode of delivery such as a drop-in appointment system, embedded peer-relational support, ability of clinical staff to prescribe, located in community locations that PWUS are familiar with and feel safe (for the full list of principles see Supplementary File S1).

The Sunderland PLUS study and the intervention were funded by the local government public health team through a grant from the Integrated Healthcare Board multiple and complex needs funding. The public health team worked in partnership with a group of primary-care practices and a lived-experience recovery organisation to deliver the bus.

The bus offered core primary healthcare services to patients, including blood-pressure monitoring, treatment of minor health conditions such as wounds or infections, providing information, advice and signposting to further sources of support. The bus was open to all adults within the target population, and others at the community venues where it was located. For patients who were not registered with a GP (general practitioner or primary healthcare doctor), they could be registered temporarily with the primary healthcare provider to access the service. The bus was operated and supervised by staff employed by the primary healthcare practices. The bus delivery team included at each session: one clinician, either a GP or Advanced Nurse Practitioner; one administrator to register and triage patients; and one service manager to oversee operational aspects of the service. The service was also staffed by one or two relational peer workers; individuals with lived experience of SUD and other multiple and complex needs, who were employed by the lived-experience recovery organisation. Peer workers were able to provide support to those attending the bus, meeting new arrivals and supporting them to register to use the bus, and providing links to accessing support for health and social needs beyond clinical healthcare. Clinical governance and safety were important considerations, and all the peer worker activities were conducted in accordance with the recovery organisation’s existing governance framework, with staff following established procedures including safeguarding, risk assessment and incident reporting. Prescribing was undertaken by appropriately qualified clinicians following national clinical guidelines within established governance and competency frameworks.

The intervention ran for six months between 1 April and 31 October 2025. Over this period, the bus visited different community organisations such as church-based hubs with a drop-in kitchen for people experiencing homelessness or food insecurity, food banks, community centres and voluntary sector recovery organisations. Factors considered when identifying possible sites included proximity to nearby referring services; established existing networks; and areas of known need relating to substance use harms or homelessness. Practically, the sites also needed to be able to meet the basic requirements of having adequate space to park the bus, space for the administration team and access to electricity and Wi-Fi to access patients’ electronic health records. At the beginning of the pilot period, sessions were held twice a week for four hours. Over the six months, a pattern was established where one session was kept at a consistent location where uptake was high, while the flexible second session was used to take the bus to different locations to establish the need and/or uptake. The service reduced to one session a week at the beginning of October 2025 due to perceived low uptake on the flexible session, but high uptake on the consistent session.

### 2.3. Study Design and Research Team

A pragmatic mixed-method process evaluation using predominantly qualitative methods, and some quantitative methods, was undertaken comprising a patient survey; overt observations undertaken by the research team at the bus sites; analysis of anonymised routine patient data for all patients who accessed the bus; and semi-structured interviews with patients who accessed the bus and staff involved in its setup or delivery. The pragmatic approach meant that data from the qualitative and quantitative methods has not been formally integrated; rather, the data is brought together to answer the research questions [46]. A pragmatic design is particularly suited to social justice research where the ambition is to generate knowledge that could lead to change for marginalised groups [47].

The research fieldwork and data analysis was conducted by four female researchers; three of these researchers had previous experience of providing care for people with multiple and complex needs but were not providing care at this site. All were experienced in conducting research with the target population. These researchers had no previous relationships with the patients and were not involved in their care. They knew most of the staff through working with them during the formative phase of the Sunderland PLUS study and the setup of the bus intervention.

### 2.4. Public Involvement and Engagement

A Patient and Public Involvement and Engagement (PPIE) group was established at the outset of the Sunderland PLUS study comprising six individuals from the region with lived experience of SUD. Some PPIE members held paid or voluntary roles within recovery services and had good knowledge of local services and issues occurring affecting this population. The PPIE group were consulted through the duration of the evaluation, meeting approximately once every two months to provide feedback across the study, including study design and delivery, development of patient-facing materials, and dissemination of findings. Additionally, one individual with lived experience of SUD joined the research team and met regularly each month to support in the delivery of the study.

### 2.5. Routine Data

Anonymised retrospective patient data was collected by the primary healthcare provider from patients accessing the bus and recorded using their electronic medical records system (EMIS). Data included demographic information, presenting health concerns, existing conditions, medical history, medications, previous use of alcohol or drugs, accommodation status, employment status, and benefits claimed. Onward referrals made by the bus practitioner were noted, but the service referred to was not recorded. For this pilot study there was no formal system in place to quantify whether patients successfully attended the referral. At the end of the study period the data from patients who accessed the bus was anonymised by the primary healthcare team and sent securely to the research team for analysis.

### 2.6. Patient Survey

The patient survey instrument (Supplementary File S2) was developed by the research team, then reviewed and edited by the PPIE group. Questions covered patients’ experiences of accessing the bus, including reasons for visiting and main health concern; their own physical, mental health and social support needs; previous recent healthcare utilisation including medications; satisfaction with the service and suggested improvements. The survey instrument also included spaces for open responses to capture any unforeseen responses. This cross-sectional survey was carried out weekly at one of the bus sites, face-to-face by the research team. Patients who attended the bus on the days the researchers were present were told about the survey either by the administrator when they registered for their appointment, or by one of the researchers themselves or a peer worker. The patients had time to consider participation in the survey and were told that participation was optional and would not affect their care on the bus. Given that substance use is common within this patient group, excluding all individuals who had consumed alcohol or drugs would have limited our study sample. We aimed to address this through use of a pragmatic approach, balancing inclusivity with careful assessment of individual patient capacity to consent at the time of recruitment. Individuals who were deemed too inebriated or unwell to be able to give informed consent (n = 2) were not approached for participation, although they were told they could complete it if they came back another week. The survey was carried out as soon as possible after the patient’s appointment on the bus with most (n = 104) being completed on the same day they accessed the service. Patients who attended the bus on days the researchers were not present were also told about the survey by the administrator or peer workers and a small number (n = 8) undertook the survey a week or two after their appointment at locations where the researchers were present. The survey took between 10 and 20 min to complete. Patients were given a £10 shopping voucher (which excluded alcohol purchases) for taking part in the survey. Patients could access the bus multiple times, but they could only complete the survey once. All survey participants were asked if they would like to leave contact details to be contacted later to participate in a semi-structured interview.

### 2.7. Overt Observations

The research team undertook observations at bus sites for a total of 30 days across the six months. Free-text observations focused on how patients were managed before consultations, e.g., in waiting areas or during triage assessments, and after consultations to establish what after-care is available, who delivers this and who accesses it. Additionally, we observed how wait times were managed, how peer workers were utilised, and how patients reacted to the triage processes, including helping identify possible reasons why potential patients may leave before they see the clinical team. Observations were not made of clinical appointments. Written observation notes were made where possible at the bus sites, with more detailed notes added the same day or the next day when the researcher returned to their desk. In addition to collecting data, it was hoped that having a researcher present at the bus site would build trust with staff delivering the service, and patients accessing it, to help promote engagement in the evaluation [48]. See Supplementary File S3 for a breakdown of time spent at each site.

### 2.8. Semi-Structured Interviews

Semi-structured qualitative interviews were carried out with patients who had accessed the bus. Most of these patient participants were recruited via the contact details they gave when completing the survey. More than half (n = 54) of the 96 survey participants who met the target population consented to give their contact details. However, 13 of those 54 people did not have a phone number or email and they gave contact details of hostels or support workers, or places researchers may come across them in community venues. Other participants (i.e., who had not first completed the survey or did not have contact details) were approached at venues which had referred people to the bus, e.g., a hostel, and a charity providing support to people experiencing homelessness. Patients were purposively sampled to ensure we included people who had accessed the bus at different sites and where possible, both men and women, recognising how gender can impact on people’s experiences with healthcare interventions [49,50]. The semi-structured interview schedule (Supplementary File S4) included questions designed to explore patients’ views about the bus and about any follow up or benefits they perceived from accessing it. The interviews were audio recorded and conducted in person at community venues (n = 12) or via the phone (n = 5). Patient interviews lasted between 10 and 30 min, and patient participants were given a £20 shopping voucher for taking part.

Semi-structured interviews with individuals or small groups were also caried out with staff involved in the set up and delivery of the bus, including primary healthcare and local government staff, peer support workers, community organisation and local recovery organisation staff. Staff were sampled to ensure inclusion of people from the different partners involved in the set up and delivery of the bus. The semi-structured interview schedule (Supplementary File S5) included questions designed to explore staff views about the aim of the bus, partnership working, challenges of delivery and perceived benefits of the bus. The audio-recorded interviews were conducted in person at the staff members’ workplaces (n = 9), via phone (n = 2) or using an online video conferencing software (Microsoft Teams) (n = 9). Staff interviews lasted between 30 and 90 min.

Recruitment for patient and staff interviews stopped when we felt we had included a sufficiently diverse range of staff and patient participant views and insights were adequately rich to meet the aims of the study [51]. The audio files from all the semi-structured interviews were transcribed by an independent transcription company and the transcripts were anonymized by the research team.

### 2.9. Data Analysis

Analysis of the data was carried out over two stages. In the first stage a deductive analysis was conducted of the individual data sets from each method, as described below. In the second stage, the quantitative and qualitative data were brought together to fit within key dimensions from the RE-AIM implementation framework. The RE-AIM framework [41,52] consists of five dimensions (Reach, Effectiveness, Adoption, Implementation and Maintenance) and is used to plan and evaluate health interventions considering not only effectiveness but also exploring adoption, implementation and long-term maintenance and sustainability of the intervention.

#### 2.9.1. Stage 1

Quantitative data from the survey and routine patient data were analysed using Excel to generate descriptive statistics exploring frequencies reporting denominators and percentages. Missing data will be reflected in the item-specific denominators.

The open responses from the survey, the interview transcripts and the observation notes were each analysed separately initially using a deductive qualitative thematic analysis with the project aims and RE-AIM framework in mind [48,53]. The research team (described above) familiarised themselves with the data by reading and re-reading the data and making notes or memos. One researcher (author 2) read and coded all the data in this way and three other researchers (authors 1, 3 and 11) coded at least 20% of the data each. The team met to discuss their deductive codes and compare notes and memos in two one-hour meetings. Similarities and differences were discussed, the final coding framework (Supplementary File S6) was agreed upon, and transcripts, open survey responses and observation notes were re-coded accordingly.

#### 2.9.2. Stage 2

In the next stage, authors 1 and 2 discussed how the codes could be condensed to fit within each of the domain descriptions from RE-AIM as detailed in Table 1. Through discussion over several one-hour meetings, they generated the sub-themes, which brought the quantitative and qualitative data together. In line with the pragmatic mixed-methods design, although the quantitative and qualitative data has not been formally integrated, there are places in the findings where the interpretation compares and contrast the data to provide a richer overview.

### 2.10. Ethical Approval

Ethical approval was granted by the NHS Health Research Authority North East—Tyne & Wear South Research Ethics Committee (24/NE/0057 IRAS 338825). Informed written or audio consent was sought from all survey and interview participants. Consent was gained from the delivery team and the venues to observe in public areas at the sites.

## 3. Results

From April to October 2025, approximately 100 h of observation were recorded at bus sites and 112 patient surveys, 17 patient interviews and 20 staff interviews were conducted. Table 2 shows the characteristics of the sample from the survey and interviews. From here we present our key findings related to the dimensions of the RE-AIM Framework, including sub-themes for each domain. Table 3 summarises these sub-themes and Supplementary File S7 summarises the supporting data captured by each method in relation to each domain. We have used anonymised participant quotes and fieldnotes as evidence to support the interpretation (see Supplementary Files S9 and S10 for additional data). Due to the small geographical area of the study, which has the potential to make participants identifiable, we have omitted detailed information about staff beyond their general role in the intervention. All survey data are reported; however, some items had missing responses, therefore denominators vary across items.

### 3.1. Reach

**Figure 1:**
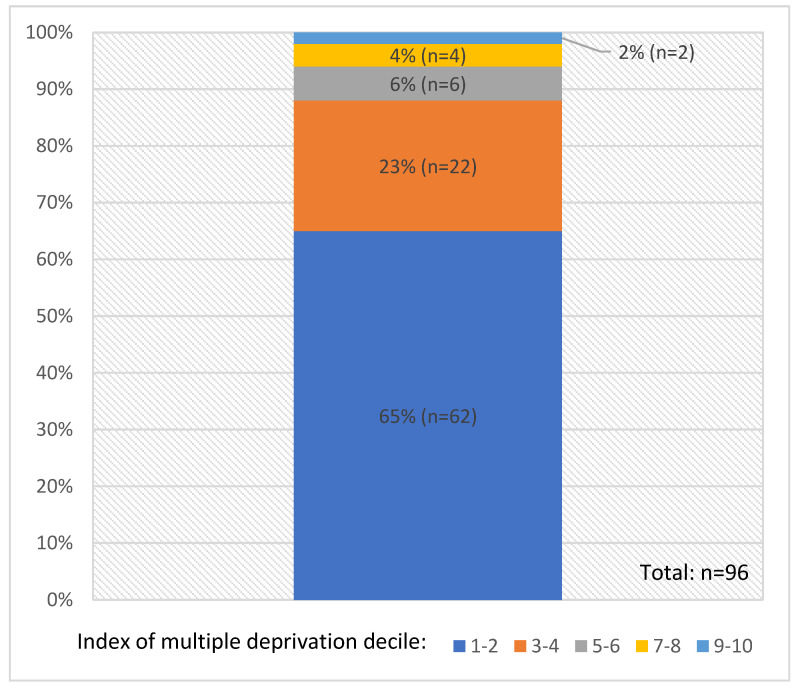
Index of Multiple Deprivation Deciles of bus patients with a home address taken from routine patient data.

The routine patient data indicated that 164 patients (115 males and 49 females) accessed the bus during the pilot period (mean age = 48 years, range 17–92). The majority (90%, n = 148) identified as white British or mixed British. Most patients met the criteria for the target population: 40% (n = 65) reported a history of alcohol use; 39% (n = 64) reported a history of drug use; and 34% (n = 55) were experiencing homelessness. Of the 96/164 patients with a home address, 65% (n = 62) resided in deciles 1–2, representing the most deprived areas nationally (see Figure 1). Routine data indicates that there were 18 onward referrals made by bus clinicians. However, the observations suggested that other onward referrals were made by peer workers that were not recorded. Additionally, the observations indicated that the primary healthcare team sometimes helped people at the bus sites who met the criteria for the target population but did not access the bus itself. For example, staff were observed supporting people with digital access to appointments for their own GP surgeries.

The majority (84%, n = 96) of survey participants were the intervention target population, as shown in Table 2. The survey data indicated that the most common presenting concerns were pain management, wounds/infections, general check-ups, medication concerns or mental ill health. Many survey participants also reported co-morbid physical or mental health conditions. More than four in five (83%, n = 99) survey participants reported pre-existing diagnosed conditions, with physical diagnoses commonly including chronic obstructive pulmonary disease, arthritis, or hypertension. Mental health diagnoses commonly included depression (73%, n = 82), anxiety (68%, n = 77), or paranoia (37%, n = 41). Patients also reported non-diagnosed mental health conditions, past trauma, and neurodiversity.

In terms of existing access to primary healthcare, 93% of survey participants (n = 104) were already registered with a GP. Despite this, 65% of participants (n = 75) reported that they struggled to access healthcare. The following extract from the observation notes are just one example of the multiple and complex needs most patients were experiencing, and further illustrate that the bus reached the target population:

*As he sat down, I could smell alcohol on him and he seemed agitated. He described using alcohol and drugs for many years to mask lifelong trauma and mental ill health, and how difficult it was to stay well so he could be there for his family. He described previously accessing [primary healthcare mental health support] for this trauma and although initially positive, once his worker told him they were changing roles and handing him to another colleague, he felt the rapport and trust had been lost and he did not continue. He said difficulties applying for access, or waiting times for assessments (for his undiagnosed PTSD) massively put him off seeking support or healthcare. Although he was registered with a GP he had not been for years. (Observations, month 2)*

Bus uptake from PWUS was highest at services specifically targeting PWUS, e.g., church-based hub with a drop-in kitchen for those experiencing homelessness or food insecurity. In contrast, other sites which provided more generic community-based services were attended by PWUS as well as individuals outside of the PWUS population.

### 3.2. Effectiveness

#### 3.2.1. Patient Satisfaction

The survey data indicated that there were high levels of patient satisfaction with the bus; 96% (n = 108) of participants rated their experience of the bus as ‘very good’ or ‘good’. Patients reported satisfaction with the practical delivery of the bus, such as immediate access, ‘drop-in’ approach, and longer appointments than standard primary healthcare where they were able to discuss multiple health issues. Patients indicated that they felt this delivery style addressed common challenges for PWUS in accessing traditional GP practices. For example, Patient 1 compared the immediate access to the bus and the time they were able to spend with the clinician to the delays in primary healthcare and perceived short appointments where they were usually only able to discuss one clinical concern:

*“What I really liked about [the bus] is if you phone up your GP for an appointment, you’re looking at [an appointment] weeks down the line, with [the bus], you’re just straight on, no appointment. Plus, if you get an appointment with your GP, it’s 10 min. On the bus, it’s half-an-hour, plus you got a full MOT”* (Patient 1, male, 50–59)

Survey and interview patient participants also reported satisfaction with the bus delivery staff, describing them as caring and welcoming, which they highlighted made them feel comfortable during appointments. Patients reported feeling listened to and thoroughly assessed by the clinician, often contrasting this with previous experiences in primary healthcare where they had not felt listened to or that their needs were not understood. Patient participants described all staff as knowledgeable, from the clinicians to the administrative team. Patient 16 and Patient 6 illustrate this feeling of being cared for, which was conveyed by other patients:

*“They were very helpful, they were making me feel calmer, asking me what questions I wanted to ask or… You know. I wasn’t nervous”* (Patient 16, female, 60–69)

*“How do I put it in words? Comfortable. Relaxed. Just going in, total stranger, and then just coming out and feeling, ‘Well… If I go to my own GP, there’s a bit of animosity, with all the other stuff that’s gone on, so basically, I haven’t been since 2018’”* (Patient 6, male, 50–59)

Patient 6’s account also further illustrates that although most patients were registered with a GP, many reported they had not accessed them for a long time.

#### 3.2.2. Re-Engaging in Healthcare

Both patient and staff participants described individual and structural barriers for PWUS engaging in healthcare, such as anxiety, lack of motivation and confidence, and fear of stigma. However, our accounts suggested that the positive experience of visiting the bus helped overcome these barriers. Patient and staff interviewees provided examples of when patients continued to engage more broadly with healthcare after their initial visit, either by re-attending the bus or re-engaging with their GP more regularly. For example, Patient 2 described that they had attended their GP more regularly for a chronic health condition since visiting the bus:

*“It’s meant I’ve kept up with my doctor every month … Because it started, that was enough initiative for me to go. Dinnit [do not] let it slip again. So, I keep in touch now with them”* (Patient 2, male, 50–59).

The staff interviews and observations suggested that structural and/or material barriers could be removed by the bus administrative team to allow PWUS access to primary healthcare. For example, by supporting people to make healthcare appointments who had no digital access, or GP registration for individuals with no fixed address. This also included the removal of historical ‘flags’ (visual markers that alert healthcare staff of potential risky behaviour) from patient records, which had prevented individuals from previously accessing primary healthcare. Staff participant 3 provided an example of this:

*“[For the patient there was information on their record] it was something like alcohol-related aggression and they’d been ‘red carded’ [banned from accessing primary or secondary healthcare] so they weren’t able to access or permanently excluded, but the [Organisation] managed to ring that practice and get that removed because it had been on there for a lot of years and obviously the situation with that individual had changed massively”* (Staff participant 3, delivery team)

Routine data indicated that 18 onward health referrals were made by the clinicians. Although we were unable to formally follow up with these referrals, some patient interviews provided insights into healthcare experiences after their bus visit. For example, Patient 4 (male, aged 40–49 years) revealed that after accessing the bus, they were driven to hospital by a peer worker for further treatment. He was recommended for surgery but felt he had to decline because he was currently living in temporary accommodation, and the staff were unable to care for him for the weeks he would recover from the operation. This is how Patient 4 explained the situation:

“Patient 4: *I thought it was a waste of time going, wasting people’s time*.

Researcher: *What, at the hospital?*

Patient 4: *Yeah, even though [the doctor] found [a health condition], he couldn’t treat me because I’ve got no one to look after me*”

This example highlights how PWUS’s wider unmet social needs can impact their engagement with/accessibility to healthcare. Despite efforts from staff to remove individual and structural level barriers, it was clear that some barriers (e.g., housing status) were too complex for the bus intervention to address alone.

#### 3.2.3. Addressing Additional Health and Social Needs

Despite the few examples where the bus could not overcome complex barriers, bus delivery staff were often able to use an interdisciplinary approach to address the multiple and complex needs that some patients presented with. They were observed working together and drawing on their individual strengths and knowledge. Peer workers were observed addressing patients’ wider needs through emotional support, understanding and shared experiences, and facilitating referral pathways into housing or substance-use support. During the observations a clinician was observed explicitly acknowledging this added valued provided by the peer workers:

*[The clinician] stood and discussed the value of the peer workers for helping patients after they had been seen on the bus. They spoke about how much work [the peer worker] did to try to help a female patient get a place in a hostel. The clinician said “If the [peer workers] weren’t there I don’t know what I could have done”. (Observation, month 3)*

**Figure 2:**
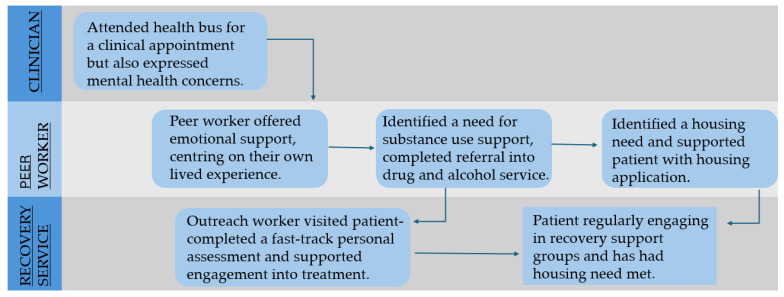
Male patient (aged 40–49 years) accessing the bus via a foodbank.

A positive example of how the interdisciplinary team supported people is also presented in Figure 2 below.

Additionally, the peer workers provided practical support to patients through transport to and from the bus and to secondary healthcare, if required. Through observation notes and staff interviews, we understood that preventative harm-reduction approaches to supporting PWUS were also being utilised; wound-care packs were available for people with wound needs, and on-site testing for blood-borne viruses was offered by one peer worker.

Host organisations were already providing support for the target population, i.e., free food, free clothes, access to hygiene and shower facilities, recovery support, social connections, or support with welfare and housing. It was noted by some staff participants that the bus effectively created a joined-up and holistic approach to health and wellbeing, acting as ‘hubs’ for the target population to adequately have their needs met in one place. Staff participant 13 highlighted this point.

*“Those who have accessed the health bus, and have been to [Venue], have found kind of a community, because it’s like, ‘I can go there, I can get fed, and get seen, I’ll meet someone I know, at least, so I’ve got a social connection,’ It’s not entirely kind of recovery focused, it is purely social, and they’re getting their health needs met”* (Staff participant 13, wider community partner).

However, although addressing these support needs can indirectly improve people’s mental health and wellbeing, several delivery staff identified a need for additional mental health support for many patients.

#### 3.2.4. Capacity Building Amongst Services

The staff interviews highlighted the potential long-term effectiveness of the intervention through capacity building in primary healthcare and improved partnership building. All participants in an administrative or clinical role within primary healthcare reported that their involvement with the bus had improved their knowledge and insights of working with the target population, an area in which they previously had limited experience. Some staff participants noted that the intervention had improved collaborative working between local drug and alcohol recovery services and community services, fostering ongoing relationships. This is illustrated in the following quote:

*“I interacted with a couple of peer recovery workers and that stimulated an ongoing relationship with [drug and alcohol service] which has been really positive… Basically, the individual who leads the [drug and alcohol service] session in [place name], they were looking for a venue, so they now meet here on a Monday. So, yeah, it’s been really fruitful.”* (Staff participant 15, host organisation staff)

### 3.3. Adoption

#### 3.3.1. Organisation’s Willingness and Ability to Be Involved

In the study region there is a well-recognised need for improved healthcare access for the target population. As a result, most (n = 7) organisations who were approached were keen and willing to take part in the pilot, as they believed that the bus would benefit their clients.

*‘So, the rest of the staff here were really quite excited about it because we could see straightaway the potential to just get people through the door, because it can really be difficult’* (Staff participant 6, wider community partner)

Some organisations who were considered or approached did not take part in the pilot. Although they supported PWUS and identified a need for healthcare, there were additional factors to consider, such as the infrastructure for a bus and administrative team to set up, and whether venues were in a ‘catchment area’ that could affect access to GP records, which were required for any bus interactions. For example, one recovery organisation did not act as a host site because they did not have space for the bus to park in their car park.

#### 3.3.2. Value of Existing Relations and Infrastructure

The support from various organisations to adopt the bus appears to have been facilitated by the pre-existing relations and cross-sector infrastructure in Sunderland. Bus administrators used an electronic medical records system that was also used within community primary healthcare, with already signed data-sharing agreements from all GP practices in the study region. As a result, the staff were familiar with the software, and no additional work was required to set it up. Having this agreement in place facilitated the ease of adoption and implementation:

*“It was easier to roll out because we didn’t have to do it from scratch”* (Staff participant 4, setup/delivery staff)

Adoption was also facilitated by well-established networks between the peer workers and local voluntary organisations, which provided initial guidance for potential bus locations.

*‘I mean, I’ve known [Peer worker] for years. She just knows we exist so she would send stuff to us and then, if there was anything different, she would let us know. Obviously, she was very much involved’* (Staff participant 9, wider community partner)

#### 3.3.3. How the Bus Was Integrated into the Wider Care Networks

Adoption of the bus as a referral option was built into routine processes for organisations through weekly email updates sent out by the bus manager regarding locations, times, and other details of the bus. By the end of the pilot there were 67 individuals across multiple organisations on the mailing list. How information was disseminated within organisations and clients depended on the organisation; for example, some who had regular team meetings or handovers were able to easily add this to their agenda. However, other staff participants felt adoption and uptake of the bus could have been improved with clearer and more prominent promotion of the bus, so that the intervention could become more embedded into their daily practice.

Initially, information about locations and times was shared from organisations to clients by word-of-mouth, but as the pilot progressed a poster was created by the primary healthcare group, which was distributed to locations that PWUS regularly accessed. Some organisations would hang posters on their noticeboard for service users to see or share electronically via social media.

Notably, the survey data indicated that the majority of the 112 participants heard about the bus by word-of-mouth from either a friend (n = 20/112, 18%) or a support or community worker (n = 31/112, 28%). In comparison, others heard about the bus from other healthcare providers (n = 4/112, 4%), poster displayed in a service (n = 8/112, 7%) or poster advertised online or social media (n = 3/112, 3%), found opportunistically (n = 32/112, 28%) or other (n = 14/112, 12%). The dominance of word-of-mouth promotion was reflected in patient interviews and illustrates that this communication was vital to uptake of the bus amongst the target population.

### 3.4. Implementation

#### 3.4.1. Clarity and Expectations of Bus Service and Staff Roles

The interview participants broadly understood the bus as providing GP access to PWUS who struggled to engage with primary healthcare. However, there was some uncertainty from staff in referring organisations and host organisations about what clinical support the bus could offer patients. Clarity about these expectations appeared to depend on how organisations shared information amongst their own teams and whether the promotion of the bus was able to become adopted into their organisation’s everyday practice. Additionally, there was some uncertainty about who the target population for the bus was, as the bus did not advertise this explicitly. This was due to a pre-implementation decision to exclude any drug or alcohol recovery services logos to the bus, as the PPIE group felt that this may stigmatise service users.

The findings highlighted a positive perception of the peer workers role and their added value. However, by the end of the pilot some patients and staff remained unclear about the role of peer workers in supporting the wider needs of patients attending the bus. Suggestions were made to improve the identification of peer workers on site including a uniform or clear identification badge. Some staff participants also felt this could have been mitigated through clearer role definition and lines of responsibility from the outset. For example, participant 4 said:

*“I think people’s roles probably could have been a lot clearer or more embedded… I don’t necessarily know … how long [peer workers] were told to stay on site or what they were supposed to do.”* (Staff participant 4, setup/delivery staff)

#### 3.4.2. Staff Qualities and Knowledge

Participants involved in the delivery and setup of the bus reported that the recruitment of clinical staff was challenging, since the specific recruiting criteria and less interest in the shift times (5 h shift, twice a week) meant a limited pool of staff to recruit from. It was apparent through the implementation that ample time and consideration was taken by the primary healthcare team to hire staff they felt met their criteria to work in the clinical or administrative roles. Experience of working with PWUS was desirable but not essential. However, having qualities that were better suited to supporting the target population and a willingness to learn was important. These qualities included empathy, a non-judgmental approach, and interpersonal skills to communicate with patients with diverse and complex needs. This was perceived as not only contributing to patient satisfaction but also helped align with the values of hosting organisations.

*“The team who work the health bus fit in really well with the community here, which is really important. We don’t want people coming in who, I don’t know, don’t have those people skills when you’re dealing with such a diverse community. That people were friendly and kind and fit in with our values of respecting other people, so that [service users] felt comfortable, that was important as well”* (Staff participant 6, wider community partner)

The observations illustrated that often quick adaptations were required by the bus delivery staff to resolve various day-to-day problems, from IT issues to specific patient queries including GP registration, prescription needs, and rescheduling appointments. The findings suggested that staff’s pre-existing knowledge and understanding of the health and care system was beneficial in such situations, Additionally, the ‘drop-in’ approach meant that appointments were generally given on a first-come basis. However, in some instances appointments needed to be reorganised based on priority of need, due to the presenting medical need assessment or whether the patient would be able to wait until their appointment. The staff were skilled at triaging and prioritising these needs and effectively communicating this with patients. The following example illustrates how the team worked together to triage patients:

*[The peer worker] sat and talked to the patient and he was joking on with her and seemed calm but visibly intoxicated. [Bus manager] had asked [peer worker] if she could sit with him and get him registered with a GP after his appointment. [Bus manager] and [administrator] bumped this gentleman up the list as others were happy to wait. (Observation notes, month 4)*

#### 3.4.3. Flexibility of Service

As a pilot project, the primary healthcare team were keen to trial the bus at different locations. In practice this was delivered by maintaining one session at the same day, time and location every week, while the other ‘flexible’ session was utilised across different days and venues. Weekly meetings between the delivery team and some community organisations were held to discuss the suitability of each location, and there was flexibility to move the bus the following week if a location was not as successful, for example with low uptake or few PWUS patients, or if there was feedback to try a different venue. Sessions were moved to earlier or later appointment times to suit the needs of the venue that the bus was attending based on feedback from host organisations or peer workers. Operational staff were responsive to the feedback and showed a willingness to adapt the intervention to meet the needs of the population. This is illustrated by Staff participant 15, who reflected that one of the earliest venues trialled had not been successful, but this was quickly reconciled:

*“Obviously, the whole purpose behind the mobility of the project… I do think it’s testament to the organisers that they, fairly quickly, said, ‘This venue isn’t working, let’s move it.’ I mean it would’ve been ironic if they didn’t move it, given it was mobile, but I think it was good that there was that kind of reaction. It was, ‘You know, let’s not drag this out too much longer, we’ve given it a few weeks now. We know where we can meet the people, let’s take it to them,’ which seems like the whole point behind it anyway”* (Staff participant 15, host organisation staff)

#### 3.4.4. Consistency

The findings indicated that consistency and familiarity were important aspects of implementation. Delivery staff became familiar with the target population at some of the host venues on a first-name basis and were thus able to offer more person-centred care based on known individual needs. Some of the target populations were already familiar to some of the peer workers. Staff provided examples of when they were able to follow up with patients regarding their health concern by attending the same venue—whether this was checking to see if they had picked up prescriptions, or to pass on information regarding their referrals into secondary healthcare. This was particularly important for patients who had no fixed address or mobile phone to contact otherwise. Although this was also completely dependent on whether the individual re-attended the venue too, which highlighted a significant challenge throughout the intervention.

Additionally, regular attendance at locations was acknowledged as having the capacity to build rapport and trust with the community. Staff explained how consistency is essential when working with the target population, as engagement with services is not always immediate, and depends on creating a safe environment in which individuals feel able to access support when they are ready.

*“But yeah, it’s just you’ve got to be there, and not ram stuff down people’s throat, and just be there when they’re ready”* (Staff participant 8, wider community partner)

Regularity of location also appeared to help the staff promote the service to their clients. Host organisations could create posters to advertise the bus service so that their clients could take away with them. But also, promotion via word-of-mouth was easier when the bus was consistent, as staff could always inform clients of its location regardless of whether they had read the weekly email update that was circulated. This was particularly useful for staff who worked in ‘outreach’ roles and were unable to check their emails regularly.

#### 3.4.5. Locations

The locations visited were in areas identified as having high needs by the peer workers with knowledge of the community. The findings highlighted the importance of the locations being already visited by PWUS, so that patients felt safe and comfortable in the environment, as well as having the bus run alongside an existing group or service, particularly if the venue was offering a free meal. This meant that there was a window of opportunity for staff to engage with the population in a seemingly relaxed environment. It also helped manage waiting times and reduce the risk of patients leaving before their appointment time, as patients could have a meal or take part in an activity while they waited.

### 3.5. Maintenance

#### 3.5.1. Funding

The staff participants’ accounts suggest that they perceived funding as the biggest obstacle for long-term maintenance, due to the instability of healthcare funding explicitly for socially excluded populations who experience multiple overlapping risk factors for poor health. This concerned not only the (lack of) availability of funding, but also considerations for any overlap with existing initiatives and how this may affect potential funding streams. Staff participant 4 described this in terms of future expansions for the bus delivering flu vaccines.

*“We could be duplicating a service that they’ve already funded within the council. So it’s not as easy as just us saying we’ll buy that flu vaccine and put it on because you have to be careful with politics”* (Staff participant 4, set-up/delivery staff)

#### 3.5.2. Long-Term Capacity and Resources

All host organisations provided water, a plug to feed the electricity cable, and a space for the administration and research team, which was considered feasible. However, some organisations expressed that they contributed more time/resources towards the intervention than initially agreed; for example, in some venues the provision of space for the administration team took up an existing room that could be used by their own staff and service users. Also, through attendance of weekly meetings, advertising in the community, or printing their own posters for clients. Although the participants expressed a willingness to contribute in this way in the short term, concerns were raised around the capacity of services to maintain this over the long term.

*‘Initially, it was just they were looking for somewhere to site the bus and then it was, “Actually, we need to plug it in. It would be really good if we could have some indoor space. Actually, can we have that space as well?” So it, kind of… I guess it evolved from just using the carpark to, kind of, taking over a large chunk of the building, which I was absolutely fine with. Very happy with that, I just felt I was probably committed before I realised what I was committed to, if that makes sense?’* (Staff participant 15, host organisation staff)

Some staff participants also outlined the high demands within their jobs; recovery workers have approximately 60 clients to support, which leaves little time to engage fully with additional initiatives. This presented as a challenge in engaging services in health interventions like the bus.

Conversations during observational fieldwork and one staff participant also considered the long-term cost of the bus itself, suggesting that the key components of the intervention could be delivered in a different way that utilised existing infrastructure. For example, by having a medical room at community venues that a clinician could deliver healthcare from. The participant felt as though this would be more cost-effective.

*“I’m not saying that people didn’t get anything out of it but, when you look at the cost of it, you think, ‘That person seeing the GP has just cost a grand (£1000).’ Do you know what I mean? If you’d stuck a nurse and a GP in a room in here, elsewhere, and had somebody accessing it once a month it would’ve been much more cost-effective”* (Staff participant 5, host organisation staff)

## 4. Discussion

This evaluation explored the impact of a relatively novel and interdisciplinary model of healthcare to improve access to primary healthcare for PWUS. Findings from the six-month co-designed pilot, mapped to the RE-AIM framework, demonstrated that the bus reached the target population as it provided access to primary healthcare and effectively engaged with PWUS. The intervention indicated effectiveness in terms of reported satisfaction in the quality of care from patients. The qualitative findings showed that some patients re-engaged with primary healthcare after a long period, attended secondary healthcare and drug and alcohol support. The intervention also helped remove historic barriers to accessing primary healthcare for some patients (red flag markers), it helped build capacity within primary healthcare in the study region for supporting the target group and it improved joint working between recovery organisations.

The project-logic model and theory-of-change statement (Supplementary File S10) illustrate the assumptions underpinning the pilot intervention, including the components, activities and contextual prerequisites, which will be needed to replicate the ‘bus’ model beyond Sunderland. The evaluation has highlighted key components and contextual features, with one of these essential components being the co-location of the bus team with a community venue that offers support for the wider social determinants of health [12]. The implementation of the bus benefited from co-locating clinical support with organisations that offer free food, social activities, benefits advice, and support with housing. Existing research has acknowledged the importance of co-locating support to create relaxed and trusted environments to better engage PWUS with healthcare [54,55]. As Pauly et al. [56] also note, it is sometimes suggested as a moral failing that PWUS do not prioritise their health. Like others, we argue that interventions to improve health equity in PWUS must recognise that people commonly need to prioritise their social needs first, and that future similar interventions should embed support to address the wider determinants of health.

Another essential feature of the intervention was the knowledge, skills and qualities of staff involved in the delivery of the service. The evaluation indicated that all members of the delivery team treated patients with respect, and without judgement or stigma, which made patients feel valued. These affective and relational dimensions of care can be overlooked in the design of healthcare services, but our findings support others who show these are fundamental for reaching this target population [14,25,28]. It is well recognised that interdisciplinary teams increase the quality of primary healthcare for PWUS, as different professionals bring different skills and knowledge to support the multiple and complex needs of patients, which extend beyond their clinical presentations [17,57]. Our study adds to this literature by demonstrating the value of experienced and remunerated peer workers working alongside primary healthcare teams. Peer workers provided important insights into the needs of PWUS, upskilled clinical staff and supported patients in accessing the bus. As well as providing them with practical and emotional support to access ongoing health and social care such as secondary healthcare appointments or drug and alcohol services. Similar benefits of peer support have been reported in other studies [58,59,60,61].

The feasibility of adoption and ongoing implementation was reliant on the existing networks and infrastructure in Sunderland. Like findings from existing evaluations of interventions combining statutory health and care providers and community organisations [54,55], the bus strengthened partnerships and built capacity to support PWUS, particularly in primary healthcare. However, a contextual feature of the study region is that prior to the intervention it already had a strong voluntary infrastructure and there was a recognised need for the intervention in the area because of the very high level of drug and alcohol related harms. Staff working in voluntary sector organisations were important for acting as host sites and for referring patients to the service. Another key contextual feature was the operational agreement between public health, primary healthcare and the lived-experience recovery organisation which enabled the project to operate as a team. Practical and technical factors were also essential, such as the space to park the bus and to set up the administrative team, and access to electricity and the internet. Moreover, access to the electronic health records ensured that the team could view the patients’ recorded medical history and add updates about the appointments on the bus for future clinical interactions.

Stigma is a contextual consideration for all healthcare interventions delivered to PWUS [62,63]. The evaluation findings suggest that the bus was delivered without interactional stigma; however, we observed the impact of structural/wider stigma on the implementation of the intervention, i.e., local service promotion and potentially the ongoing maintenance of the service [56,62]. During the co-design of the intervention, there were concerns that advertising the service in the wider community as explicitly for PWUS might lead patients to feel stigmatised if they were known to be accessing a bus for PWUS. Additionally, there were concerns that if the wider community knew about the intervention, they may question whether the target population ‘deserved’ the additional resource. This lack of clear advertising of the bus appears to have contributed to concerns raised about the lack of clarity about who the bus was for and what it could provide.

### 4.1. Strengths and Limitations

A key strength of the study was the co-design approach to the intervention and evaluation. Moreover, the qualitative methods ensured the findings were grounded in the lived experiences of the target population and allowed us to explore the wider context of care provision in the study locale [64]. The evidence about the implementation and future maintenance of the bus would not have been generated via the other methods. The pragmatic mixed-method study design allowed for triangulation and a greater validity of the data [48]. The range of methods and experienced research team helped to capture the views of a variety of participants, including the most marginalised individuals who are often excluded from research [65].

The seasonal timings of the intervention could be considered a limitation of the study. Since the pilot was predominantly delivered during Spring and Summer, the study was unable to explore the potential seasonal differences that may have been experienced in Autumn and Winter, where health and social needs differ due to the colder weather [66]. Also, the routine data only revealed the patients’ first time of accessing the bus; therefore, we cannot quantify the number of patients who accessed the bus more than once. However, our interviews and observations do show that re-attendance was common.

Another limitation was the recording of onward referrals. Clinical referrals from the routine data did not specify which service the referral was for, i.e., primary healthcare, secondary healthcare or other community services. Referrals made by non-clinical staff, including supported self-referrals, were not formally recorded. As a result, the reported 18 onward referrals are likely to be an underrepresentation of support provided. Additionally, we recognise that factors such as gender, class, ethnicity and sexuality can impact people’s experiences with healthcare, both individually and in tandem to each other [67]. The study lacked a specific reference to intersectionality, particularly ethnicity, due to the predominantly white population in the study region and at venues where the bus was located.

### 4.2. Recommendations for Future Interventions for the Study Target Population

The study evaluation indicates that future similar interventions would benefit from more clearly defined roles and responsibilities amongst the team from the outset. Findings also indicate that mental ill health was highly prevalent in the patient cohort. Future similar interventions should try to embed more mental health support. The team should also keep a referral log, and record referrals to other services, e.g., mental health care, so patients can be followed up to see if they attended referrals and try to reengage patients if they do not attend.

Beyond the bus intervention itself, the evaluation highlights socio-cultural factors such as stigma and systemic barriers which a downstream intervention alone cannot address. The ultimate goal should be to eliminate the upstream causes of SUD such as poverty, including poor housing/homelessness, and trauma. In the medium and short term there is an urgent need at policy level evidence-based measures, which we know can reduce alcohol and drug related harms [3]. Public health teams need to continue to promote evidence-based primary preventative interventions in primary healthcare and other settings such as alcohol brief interventions [3,68]. System-level initiatives should be considered to upskill the primary and secondary healthcare care workforce in working with PWUS [69,70], including training to reduce stigma such as the ‘recovery ally’ training delivered in this project. At a system level there also needs to be greater recognition that the move towards digital access in healthcare is already increasing inequalities for PWUS [71], with a clear plan to support digital inclusion. There is also a need for a system-level approach which targets pathways from primary to secondary healthcare for common conditions found in the population such as liver disease [72].

### 4.3. Recommendations for Future Research

Full-scale evaluations of similar initiatives should collect routine data related to a quantitative measure of reengagement with primary healthcare. They should also record referrals by both peer workers and clinicians, and implement a means, e.g., data linkage, to track referrals from the intervention to other secondary healthcare services, voluntary sector organisations, drug and alcohol services and other social care. We know that gender, age, ethnicity, and sexuality will affect engagement with such an intervention, and future studies should stratify the analysis by these factors, particularly considering the intersecting determinants that amplify disadvantage. A better understanding of these structural influences on healthcare access can inform future health policy for marginalised populations. Future studies should also stratify the analysis by housing status, i.e., homeless and housed, as we know people who are homeless may face additional barriers to access. Stratification of the data by different sites/locations is also recommended to enable more detailed understanding about the reach of the programme and effectiveness at different sites. Moreover, future studies carried out over a longer period should incorporate seasonality sensitivity checks to support qualitative evidence that outcomes are due to the intervention. This could also help to understand the health needs at different times of the year and to predict future use of interventions. There is social return on investment and economic evaluation currently being finalised for this study. However, findings will only be indicative, as a longer evaluation (at least one year) would be needed for definitive economic analysis.

## 5. Conclusions

This study draws attention to the complexities of healthcare presentations for PWUS and further evidences the need for, and value of, new models of care that reflect the compounding health and social needs of the population. Flexible interdisciplinary approaches that utilise relational support have the potential to address these social determinants of health alongside clinical care. Approaches should also focus on building strong community networks to improve multi-agency working, as this has the potential to improve healthcare access for PWUS. However, while innovative models of care may mitigate some of the intersecting challenges faced by PWUS, persistent structural barriers require coordinated policy reform to address the social determinants of health through primary prevention, and systemic barriers to accessing equitable care.

## Figures and Tables

**Table 1:** Description of the RE-AIM domains [53] and how it was interpreted in this project.

Domain:	Definition	Data Source
Reach	The absolute number, proportion, and representativeness of individuals who are willing to participate in a given initiative, intervention, or programme.	Number, demographic characteristics, and health needs of patients who accessed the bus (survey, routine data, interview and observation data)
Effectiveness	The impact of an intervention on important outcomes, including potential negative effects, quality of life, and economic outcomes.	Satisfaction/engagement with and perceived impact of the bus (survey, routine data, interview and observation data)
Adoption	The absolute number, proportion, and representativeness of settings and intervention agents (people who deliver the programme) who are willing to initiate a program.	Number and characteristics of professionals and settings involved in delivery of the bus. Willingness and capacity of professionals and settings to be involved. (survey, interview and observation data)
Implementation	At an individual level, clients’ use of the intervention strategies. At a setting level, the fidelity to the various elements of an intervention’s protocol, including consistency of delivery as intended and the time and cost of the intervention.	Adaptions made and challenges faced during the delivery of the intervention. Consistency of implementation across settings/time/staff/population. (interview and observation data)
Maintenance	The extent to which a programme or policy becomes institutionalised or part of the routine organisational practices and policies.	Factors influencing continuation or discontinuation of the intervention. (interview and observation data)

**Table 2:** Survey and Interview Participant Characteristics.

Patient Survey	Number (%)
Gender:	
Male	77/112 (69%)
Female	35/112 (31%)
Age range	23–87 years
Mean age	48 years
Ethnicity:	
White British	110/112 (98%)
White non-British	2/112 (2%)
Meeting the PWUS population/study criteria:	
Met PWUS criteria/target population criteria	96/112 (84%)
Did not meet PWUS criteria/target population criteria	18/112 (16%)
In recovery from SUD	56/109 (51%)
Use of illicit drugs (over agreed cutoff *)	35/112 (31%)
Use of alcohol (over agreed cutoff **)	35/112 (31%)
Experience of homelessness (past or current)	67/110 (61%)
**Staff Interviews**	
Intervention role:	
Planning and set-up	2
Delivery	5
Host venue	5
Wider community partners	7
Setup/Delivery	1
**Patient interviews**	
Gender:	
Male	12/17 (71%)
Female	5/17 (29%)
Age range	29–69 years
Mean age	49 years
Ethnicity:	
White British	15/17 (88%)
White Other	1/17 (6%)
Black/African/Caribbean/Black British	1/17 (6%)

* In the past 3 months, used illegal or non-prescribed substances either daily or once per week. ** In the past 3 months, consumed more than 14 units of alcohol per week either every week or every other week.

**Table 3:** Summary of analysis themes and sub-themes.

Reach	
Effectiveness	Patient satisfaction Re-engaging with healthcare Addressing additional health and social needs Capacity building amongst services
Adoption	Organisations willingness and ability to be involved Value of existing relations and infrastructure How the bus was integrated into the wider care networks
Implementation	Clarity and expectations of bus services and staff roles Staff qualities and knowledge Flexibility of service Consistency Locations
Maintenance	Funding Long-term capacity and resources

## Data Availability

The datasets presented in this article are not readily available because of the small and identified study area which would make participants potentially identifiable. Requests to access a summary of the coding frameworks and associated data extracts should be directed to the corresponding author.

## Supplementary Materials

The following supporting information can be downloaded at: https://www.mdpi.com/article/10.3390/healthcare14050670/s1. Supplementary File S1—Sunderland PLUS Study Co-designed Intervention Principles; Supplementary File S2—The Patient Survey Instrument; Supplementary File S2—Table of observations time spent at sites; Supplementary File S4—Patient Interview Topic Guide; Supplementary File S5—Staff Interview Topic Guide; Supplementary File S6—Stage 1 qualitative data coding framework; Supplementary File S7—RE-AIM data table; Supplementary File S8—Additional interview data; Supplementary File S9—Additional observation data; Supplementary File S10—Logic model and theory of change statement.

## Abbreviations

The following abbreviations are used in this manuscript:

PWUS	People who use substances/with a substance use disorder
SUD	Substance use disorder
NHS	National Health Service
GP	General Practitioner/primary healthcare doctor
PPIE	Patient and Public Involvement and Engagement
