# Supplementary material for: Findings from the Process Evaluation of a Mobile Health Clinic Designed to Improve Equity of Access to Primary Healthcare for People with Substance Use Disorders and/or Homelessness in One Region in the North East of England, UK
Source: Healthcare (Basel). 2026 Mar 6;14(5):670. doi: 10.3390/healthcare14050670 (PMC12985337; doi:10.3390/healthcare14050670)
Supplement: Supplementary file 1 [file healthcare-14-00670-s001.zip › healthcare-4125533-supplementary/Supplementary S1 - Intervention design principles.pdf]

Supplementary S1 - Sunderland PLUS Study Co-designed Intervention Principles

1. Recognise that people are relational – they need to be supported and need to feel cared for
2. Take a holistic approach where healthcare sits alongside other support considering the individual and the wider context including social determinants e.g. access to food banks or housing advice.
3. Recognise the value that peer workers with lived experience of alcohol or substance use can bring to service delivery, particularly around engaging with the population
4. Learn from and build on individuals and assets within community and voluntary sector organisations, including the recovery community to ensure healthcare staff are empowered to be facilitators rather than controllers of individuals' recoveries
5. Need to recognise adverse life experiences and trauma
6. Recognise and accommodate additional challenges created by mental ill health, neurodiversity, gender, race, culture or sexual orientation.
7. Through training and awareness raising, address and counter the basic care sometimes experienced in statutory healthcare and build on the examples of good care.
8. Challenge stigma and promote visible recovery - Encourage a culture of collaboration and mutuality recognising that everyone has a role to play in providing safe, non-judgmental, and recovery-oriented support
9. Maintain an environment that fosters empowerment, voice, and choice, while reducing the burden of responsibility and impossible expectations weighted on the PLUS population and unpaid carers
10. Keep things simple. Make accessing and navigating services easier for people.
11. Keep communication transparent, to foster relationships built around trust so 'windows of opportunity' can be utilised.
