# Supplementary material for: Findings from the Process Evaluation of a Mobile Health Clinic Designed to Improve Equity of Access to Primary Healthcare for People with Substance Use Disorders and/or Homelessness in One Region in the North East of England, UK
Source: Healthcare (Basel). 2026 Mar 6;14(5):670. doi: 10.3390/healthcare14050670 (PMC12985337; doi:10.3390/healthcare14050670)
Supplement: Supplementary file 1 [file healthcare-14-00670-s001.zip › healthcare-4125533-supplementary/Supplementary S10 - Logic Model and Theory of change statement.pdf]

Logic model for a mobile health clinic offering primary care without stigma in the community for people with substance use disorders with and without homelessness (referred to as 'the PLUS population')

RESOURCES / INPUTS

Clinician who can prescribe (GP or Advanced nurse practitioner)

Peer workers with lived experience of substance use disorder and or homelessness

Administrator, project manager, IT support, Driver

Mobile health clinic

Vehicle-accessible community venue, that is already providing support to PLUS population

Access to electronic medical records system, standard operating procedures, data sharing agreements

Support of other community organisations who provide support to PLUS population for referrals

ACTIVITIES

Training for clinicians in being a 'Recovery Ally' stigma free.

Advertise and promote the services to community organisations and PLUS population

Register patients for a bus appointment and triage patients based on observed need

Provide stigma free and accessible primary care to the PLUS population

Provide non-judgemental compassionate relational support to PLUS population at community venue

Provide navigational support to PLUS population to access drug and alcohol treatment, and support with social determinants as needed

Provide support for PLUS population to access future primary care appointments i.e. register with a GP

MECHANISMS

Patients:

Experience service as being stigma free

Experience relational peer support to access the bus

Experience navigation support to access drug and alcohol treatment, services that can help with social determinants (e.g. food banks, housing and benefits advice) and future healthcare appointments

Positive interactions lead them to feel valued / improved self esteem

Staff / workforce:

Have increased confidence working with PLUS population

Cognisant of adverse life events and trauma

Providing a relational approach to support (e.g. listening, validating, non-judgemental)

Outcomes

Reach

- Accessed by the PLUS population with unmet physical and mental health needs

Effectiveness

Patients

- Improved PLUS patient experience of primary care
- Early treatment for health conditions (particularly wounds, infections, and injuries) (Tertiary prevention)
- Follow-up of unmet preventative interventions e.g. cervical smear tests, breast checks (secondary prevention)
- Patients accessing support for alcohol and drug dependency (secondary prevention)
- Patients accessing support for social determinants of health e.g. housing, food banks (primary prevention)

Staff / workforce

- Improved confidence working with the PLUS population
- Improved confidence and experience working with a clinician

System level

- Primary care more responsive to specific health care needs of PLUS population
- Improve working relationships and partnership working between primary care, drug and alcohol services, local authority and community organisations

Adoption

- Community organisations willing to host the mobile health clinic

Implementation

- Core components needed for the implementation of similar interventions identified e.g. clear role planning, staff skills, flexible and responsive delivery

Maintenance

- Core components needed for the future maintenance of similar interventions identified e.g. funding

CONTEXTUAL PRE-REQUISITES: Funding, access to electronic medical records systems, tenacious and experienced project manager and administrators, experienced lived experience peer workers to provide, advocacy, outreach, signposting and practical and emotional support for patients and information for practitioners, Recovery approach, System co-ordination, Sensitive to intersectionality (neurodiversity, ethnicity, gender), Strong links with VCSE services who can advocate for patients , Strong links with recovery community who can support patients , Capitalise on community assets (e.g. VCSE), Access which does not require digital methods/simplified booking system, Clinical staff who as stigma free and understand the needs of the population

Theory of change statement:

Providing primary care in a community setting where the PLUS population feel comfortable and trust the staff in the community setting, will make it easier for the PLUS population to access clinical services and will provide a window of opportunity to increase levels of engagement with healthcare. If the service is experienced as stigma free, trauma-informed, and non-judgemental, the PLUS population will access the bus and get the help for acute conditions earlier than they would otherwise have done, preventing conditions becoming worse. Relational peer supporters working alongside the clinical team will help to encourage the PLUS population to access the 'bus' by creating a safe and caring delivery environment. The relational peer supporters will provide navigation support and where appropriate encourage access of recovery support, and link with food banks, housing and benefits advice. Receiving stigma free care will help the PLUS population to feel positive about themselves (and valued) and support future autonomy.
