# Supplementary material for: Findings from the Process Evaluation of a Mobile Health Clinic Designed to Improve Equity of Access to Primary Healthcare for People with Substance Use Disorders and/or Homelessness in One Region in the North East of England, UK
Source: Healthcare (Basel). 2026 Mar 6;14(5):670. doi: 10.3390/healthcare14050670 (PMC12985337; doi:10.3390/healthcare14050670)
Supplement: Supplementary file 1 [file healthcare-14-00670-s001.zip › healthcare-4125533-supplementary/Supplementary S2 - The patient survey instrument.pdf]

Supplementary S2: The patient survey instrument

Participant ID:

Researcher: LT / EH / KJ / Other (circle)

Bus Location:

Date patient attended bus:

Date patient completed survey:

Input onto database on:

Sunderland PLUS Patient Evaluation Survey

This survey asks you for some personal details about yourself and your health, so that we can find out who is using the health bus and understand what the staff on the bus have helped you with today. We will also ask about how happy you are with the treatment you received today.

You might have been asked some of these questions by the Doctor or Nurse already today. This is because the appointment you had was confidential and anything you told them is kept between yourself and the Doctor/Nurse and not shared with us as researchers. Likewise, we won't share anything you tell us in the survey with the staff on the bus.

We expect this survey to take about 15 minutes, it might take more or less time depending on how much you have to say. You do not have to fill this out, and if you choose not to, it will not affect the service you receive today or will receive in the future. You can choose to skip questions you don't want to answer. Your name is not used in this survey, as we give you a random participant ID.

Section 1: About your visit today

The next few questions ask you about your experience visiting the bus today.

*1. What was the main health issue you wanted to get help with today?

Do you feel like you got the help you wanted for [insert condition]?

1. Yes
0. No

If no please tell us why…

Did you get help for anything else today?

*2. Who did you speak to today?

1. Doctor
2. Nurse
3. Peer supporter

*3. How long did you have to wait to be seen today (in minutes)?

*4. Were you happy to wait this long?

1. Yes
0. No

*5. How did you find out about the Bus?

1. Friend or family member
2. Another healthcare provider
3. Support or community worker
4. Poster/ advert at a community venue
5. Advert online/social media
6. Opportunistic/walking by
7. Other…

*6. Thinking about your visit to the bus today overall, how was your experience of our service?

5. Very good
4. Good
3. Neither good nor poor
2. Poor
1. Very poor

*7. Please tell us anything we could have done better?

1. Location
2. Open times
3. Wait times
4. Staff
5. Links to the community
6. The bus set up or layout
0. Nothing
7. Something else…

Please give any details

8. Have you spoken to a health professional before your visit to the bus about [insert health concern]?

1. Yes
0. No

If yes, who did you see?

1. Doctor
2. Nurse
3. Pharmacist
4. Other

If yes, thinking about that visit overall, how was your experience of the service?

5. Very good
4. Good
3. Neither good nor poor
2. Poor
1. Very poor

Section 2: Your health and previous experiences of healthcare

The next questions ask about your health and experiences of healthcare more generally.

9. In general, would you say your health is…

1. Poor
2. Fair
3. Good
4. Very good
5. Excellent

*10. Do you have any diagnosed physical or mental health conditions?

1. Yes
0. No

If yes, please give any details you are happy to share (for example the name of the condition or how long you have had it)

*11. Do you have any health concerns (physical or mental) that haven't been formally diagnosed?

1. Yes
0. No

If yes please give any details you are happy to share.

*12. Do you currently use any prescribed medications?

1. Yes
0. No

If yes, please state:

*13. Have you ever had problems with the following physical health conditions:

1. Blood pressure
2. Blood sugar
3. Cholesterol levels
4. Heart
5. Lungs or respiratory
6. Liver
0. None

Please add in details

14. In the past year, have you experienced any of the following mental health concerns:

1. Depression
2. Anxiety
3. Paranoia
0. None
4. Other mental health concern? If yes please state:

*15. Are you currently, or have you in the past, been in recovery from alcohol or substance use?

1. Yes
0. No
2. Rather not say

*16. How often in the last 3 months have you consumed more than the recommended 14 units of alcohol per week? (estimated at 6 glasses of wine or 6 pints of beer).

1. Every week

2. Every other week
3. Once a month
0. Never

*17. How often have you used illegal or non-prescribed substances in the past 3 months?

1. Daily
2. At least once a week
3. At least once a month
4. Once
0. Never

*18. Are you registered with a GP?

1. Yes
0. No
2. Not sure

If no, were you registered on the bus today?

1. Yes
0. No
2. Not sure

*19. Before today, when was the last time you had a GP appointment for your own health?

1. This week
2. This month
3. In the last 6 months
4. In the last year
5. 1-3 years ago
6. Over 3 years ago

*20. Have you been to A&E in the last year, for you own health?

1. Yes
0. No

21. Have you been admitted to hospital in the past year?

1. Yes

0. No

*22. Have you ever found it hard to get the healthcare you needed?

1. Yes
0. No
2. Not sure

If sometimes or yes, why?

1. Transportation issues
2. Previous bad experiences
3. Not allowed to access services
4. No appointments
5. Change of address or no fixed address
6. Feeling judged/ stigma
7. Other…

<u>Section 3: About you</u>

These next questions ask for some details about yourself so that we can report who has been using the service.

*23. How old are you?

*24. What is your gender?

1. Man
2. Woman
3. Non-binary
4. Other
0. Prefer not to say

*25. What is your ethnicity?

1. White
2. Mixed/Multiple ethnic groups
3. Asian/Asian British

4. Black/ African/Caribbean/Black British
5. Other ethnic group, please specify________________________________
0. Prefer not to say

*26. Where do you live? Please give your postcode, or if you don't currently have a fixed address, you can give the area where you are staying at the moment.

*27. Are you currently or have you in the past experienced homelessness?

1. Yes
0. No

*28. What is your current income situation? Please tick all that apply.

1. Employed
2. Retired
3. Student
4. Employment benefits
5. Disability benefits
6. Other…

*29. Do you have any caring responsibilities? For example, looking after a partner or relative. Please give any details.

1. Yes
0. No

30. Have you ever accessed any of these local services? Please tick all that apply.

- Recovery Connections
- CGL/Wear Recovery
- NERAF
- Alcoholics Anonymous
- Narcotics Anonymous
- Wearside Women in Need
- Wise group
- Mind
- Sunderland Soup Kitchen
- Micky's Place
- ELCAP
- Back on the Map
- Swan Lodge

- Poplar House

31. People sometimes look to others for companionship, assistance, or other types of support. How often is each of the following kinds of support available to you if you need it? Choose one

Someone you can count on to listen to you when you need to talk

0. None of the time
1. A little of the time
2. Some of the time
3. Most of the time
4. All of the time

Someone to give you information to help you understand a situation

0. None of the time
1. A little of the time
2. Some of the time
3. Most of the time
4. All of the time

Someone to take you to the doctor if you needed it

0. None of the time
1. A little of the time
2. Some of the time
3. Most of the time
4. All of the time

Thank you for completing this survey

Further research

As part of this research, in four-six weeks time, we would like to speak to a smaller number of people in more detail about their experiences of health and health care and about accessing the bus. This would involve an informal interview, that would take about 30 minutes, with the researcher at a time and place convenient to you.

Are you interested in taking part in further research on the topic of the bus and health care in Sunderland. Participants will be offered a £25 voucher for taking part in the one to one interview.

❍ Yes, I would be interested in taking part in further research (provide PIS)
❍ No, I would not be interested in taking part in further research

---

If Yes, what are your contact details? (for further research)

Name ____

Telephone Number ____

Email address ____
