# Supplementary material for: Findings from the Process Evaluation of a Mobile Health Clinic Designed to Improve Equity of Access to Primary Healthcare for People with Substance Use Disorders and/or Homelessness in One Region in the North East of England, UK
Source: Healthcare (Basel). 2026 Mar 6;14(5):670. doi: 10.3390/healthcare14050670 (PMC12985337; doi:10.3390/healthcare14050670)
Supplement: Supplementary file 1 [file healthcare-14-00670-s001.zip › healthcare-4125533-supplementary/Supplementary S3 - Observations table.pdf]

Venues	Average day on site (varied depending on how busy the site was with some days being shorter)	Days operating with researchers present	Total hours at each site
Venue 1	8.30-12.30	10	41
Venue 2	9.39-1.30	6	26
Venue 3	9.30-1.30	6	25
Venue 4	10.30-2.30	2	8
Venue 5	9.30-1.30	1	4
Venue 6	9.30-1.30	2	8
Venue 7	10-1	1	3
		Total days: 28	Total hours on site: 115
