# Supplementary material for: Findings from the Process Evaluation of a Mobile Health Clinic Designed to Improve Equity of Access to Primary Healthcare for People with Substance Use Disorders and/or Homelessness in One Region in the North East of England, UK
Source: Healthcare (Basel). 2026 Mar 6;14(5):670. doi: 10.3390/healthcare14050670 (PMC12985337; doi:10.3390/healthcare14050670)
Supplement: Supplementary file 1 [file healthcare-14-00670-s001.zip › healthcare-4125533-supplementary/Supplementary S4 - Patient Interview Topic Guide.pdf]

Research study: Sunderland PLUS Bus evaluation

Sunderland PLUS Patient Participant Follow up Interview Topic Guide

- Review the participant information sheet and allow for any queries or clarifications.
- Ensure consent form is completed
- Explain - Thank you for taking part in this research to better understand your experience of the Sunderland PLUS Bus and how you have been getting on since your visit. We will use the findings of the research to help us to improve the PLUS bus. There are no right or wrong answers we are interested in your views and experiences.
- Reiterate issues of confidentiality and anonymity and what is going to happen to the data.
- Check happy to record and switch on audio recorder (if consented)
- Complete Personal Details form at end or beginning of interview

Keep in mind:

- Making it a safe space - only share what comfortable with
- De-brief sheet

Section 1: Ahead of bus visit ***researcher look back over survey and provide brief overview/prompt about their visit**

1. I'd like to hear about your visit to the PLUS Bus. Could you tell me why you wanted to visit?
2. What encouraged you to visit? A friend/support staff etc?
3. How does the bus compare with other healthcare settings e.g. GP service or pharmacy?
    a. What was different?

Section 2: While visiting bus

4. Can you describe what happened when you arrived at the bus?
    a. How did you feel before visiting?
5. Were you given treatment for the concern you went to the bus for? E.g. if chest infection was main concern, was this treated?
6. Were any other issues or concerns talked through on the day? Could you describe these?

7. To what extend did your visit to the bus lead to you doing anything differently? E.g. accessing a service, getting registered with a GP, change in medication, being more likely to return to that particular community venue?

Section 3: After the bus visit

8. Did anyone speak with you while you were waiting to go on the bus or afterwards?
    a. Peer worker
    b. Organisation staff or volunteers e.g. NERAF worker
9. Did anyone talk about making a referral for you for anything? Can you tell me about that?
10. If mentioned referral before, has this been followed up? Can you describe what happened next?

Section 4: Interactions and setting

11. How did the staff make you feel? – safe/anxious/judged
12. How did the location/environment make you feel? – safe/anxious/judged
13. How can we make future patients feel safe when they visit?

Section 5: Overall feedback

What did you like about your PLUS Bus experience?

What did you think of the peer worker? What did they help you with?

What did you dislike about your PLUS Bus experience?

How can we improve the PLUS bus?

What do you remember most about your visit to the bus?

Would you recommend the PLUS bus to a friend?

That was the last question.

Is there anything else you would like to add? Thank you

Debrief
