# Supplementary material for: Findings from the Process Evaluation of a Mobile Health Clinic Designed to Improve Equity of Access to Primary Healthcare for People with Substance Use Disorders and/or Homelessness in One Region in the North East of England, UK
Source: Healthcare (Basel). 2026 Mar 6;14(5):670. doi: 10.3390/healthcare14050670 (PMC12985337; doi:10.3390/healthcare14050670)
Supplement: Supplementary file 1 [file healthcare-14-00670-s001.zip › healthcare-4125533-supplementary/Supplementary S5 - Staff Interview Topic Guide.pdf]

Sunderland PLUS Research study: Phase 2 Evaluation

Sunderland PLUS Staff Participant Interview Topic Guide

- Review the participant information sheet and allow for any queries or clarifications.
- Ensure consent form is completed
- Explain - Thank you for taking part in this research to better understand their role in the Sunderland PLUS Bus and their thoughts on the intervention. We will use the findings of the research to help us to improve the PLUS bus. There are no right or wrong answers we are interested in your views and experiences.
- Reiterate issues of confidentiality and anonymity and what is going to happen to the data.
- Check happy to record and switch on audio recorder (if consented)
- Complete Personal Details form at end or beginning of interview

Keep in mind:

- Making it a safe space - only share what comfortable with
- De-brief sheet

Background info

1. Can you tell me your job title and how long you have worked in that sector?
2. Can you tell me about your experience of working with individuals affected by alcohol or substance use, or homelessness?
3. What has your role been in relation to the bus? Set up, delivery, wider services?
4. Can you tell me about how you came to be involved in the health bus?
5. What were your understanding of the aims of the bus?

Pre-implementation *for those involved in set up

6. Can you tell me about your role and what was involved on a day to day basis in relation to the bus?
7. What was needed to get the bus set up?
    a. Location/drivers/equipment/agreements/partnership working

What were the main difficulties you experienced during set up? What helped?

8. Prior to implementation of the bus, what were your hopes for treatments available/clinical pathways/staff? E.g. treatment or clinical pathways available, staff who may be present?
9. Is there anything different or unique about this service?
10. Thinking about the set-up of the bus, has there been any difference in the way you usually work?
11. Do you think the PLUS bus is meeting the health and wider needs of the PLUS population and why do you think that?

During roll out

12. The bus has been trialed at a number of venues. Thinking back on when and where the bus started and how things have changed, can you tell me what you think has worked well?
13. To what extent do you feel the bus is meeting the needs of those affected by alcohol, substance use or homelessness?
    a. Health needs
    b. social needs
    c. recovery needs
14. Whap aspects of the health bus do you feel help to meet those needs?
15. What enables you to do your role with the bus? Can you describe the challenges you have faced in your role with the bus?
    a. Time keeping
    b. Confidence in role
    c. Collaborative working
    d. Roles and responsibilities

What do you think are the wider challenges for the health bus?

    e. Locations
    f. Staffing
    g. Research – vouchers?

16. The bus relies on working closely between services. Can you tell me about your experience of engaging with wider services? How do you feel the bus has been received?
17. We know that patients on the bus have been referred to different places during their visit e.g. hospitals, crisis team etc. What capacity do you feel local health and

social care services have at the moment to meet the ongoing needs of this population?

Future considerations

18. From your perspective, how do you feel about the PLUS bus as an approach to meet the needs of the PLUS population?
19. Do you have any specific examples of when you have seen the bus serving this population well?
20. Do you have any specific examples of when the bus has not been able to meet people's needs?
21. Do you have any thoughts on how the bus could be improved?
22. What do you think might be barriers to the long-term continuation of the PLUS bus?
    a. *prompt funding/partnership working/
23. On the other hand, what factors might support or facilitate the PLUS bus in the long-term?

Is there anything else you want to say about the bus that you haven't had the chance to?

Thank you for your time.
