# Supplementary material for: Findings from the Process Evaluation of a Mobile Health Clinic Designed to Improve Equity of Access to Primary Healthcare for People with Substance Use Disorders and/or Homelessness in One Region in the North East of England, UK
Source: Healthcare (Basel). 2026 Mar 6;14(5):670. doi: 10.3390/healthcare14050670 (PMC12985337; doi:10.3390/healthcare14050670)
Supplement: Supplementary file 1 [file healthcare-14-00670-s001.zip › healthcare-4125533-supplementary/Supplementary S6 - Stage 1 qualitative data coding framework.pdf]

Stage 1 - Theme codebook.

**Reach:**	Describe sample and its heterogeneity.
	Demographics – presenting health needs / other conditions, deprivation, etc.
	Engagement with healthcare – not just about being registered with a GP – other barriers.
	Advertising / stigma.
**Effectiveness:**	From PLUS perspective - how did they feel – sense of value, safe in environment – peer worker for reassurance.
	From PLUS perspective – the delivery of the intervention, e.g. they liked the longer appointments so they could speak openly and be listened to.
	From [healthcare provider] perspective they removed systemic barriers e.g. people with flags on system, digital exclusion.
	Reengaging in primary healthcare.
	Next step – benefit of bus/peer worker in addressing next steps linked to social determinants or mental health
	Capacity building in primary care
	Improved partnership building – broadened partnerships - particularly with recovery services
	What was the value to the locations?
	Referrals (routine)
**Adoption:**	Buy in was easy as known problem among services
	Value of existing relations
	Concerns from some site who didn't want to adopt bus – identifying service users/comprising trust, harder for services on border – issues accessing medical records if out of area
	General service vs targeted to PLUS
**Implementation:**	Intervention development led by PPIE identified needs and priorities – staff prescribing, weekly attendance of bus, advert without stigma, drop in.
	Clarity and expectations of bus service.
	Clarity and expectations of peer workers
	Staffing – Qualities (empathy, kindness) and knowledge of staff (understand healthcare system) vs practicalities of having shifts covered etc.
	Staffing - Operational skills: e.g. Able to triage and prioritise needs and communicate this with others effectively
	Access to information systems
	Flexibility/long game (time to know community, build rapport and trust) to provide person centred care which reflects the heterogeneity of the population and wide range of needs.

	Locations – targeting PLUS but also looking at wider general community venues e.g. [Venue] but in areas identified as having local PLUS needs informed by peer workers/lived experience.
**Maintenance:**	Capacity/responsibility – roles and responsibilities of staff (relational work- identifying patients to be seen, calling local services to identify need), and infrastructure.
	Could recovery peer workers take role of identifying potential patients and co-ordinating? Or champions within each service to lead on health bus engagement?
	Funding and evidence.
	Resources
