# Supplementary material for: Findings from the Process Evaluation of a Mobile Health Clinic Designed to Improve Equity of Access to Primary Healthcare for People with Substance Use Disorders and/or Homelessness in One Region in the North East of England, UK
Source: Healthcare (Basel). 2026 Mar 6;14(5):670. doi: 10.3390/healthcare14050670 (PMC12985337; doi:10.3390/healthcare14050670)
Supplement: Supplementary file 1 [file healthcare-14-00670-s001.zip › healthcare-4125533-supplementary/Supplementary S7 - RE-AIM data table.pdf]

RE-AIM data table

	Routine patient data	Survey data	Qualitative interviews	Observational fieldwork
**Reach:**	40% (n=65) reported a history of alcohol use; 39% (n=64) reported a history of drug use; and 34% (n=55) were experiencing homelessness.<br><br>Of the 96 patients with a home address, 65% resided in deciles 1–2, representing the most deprived areas nationally.	84% (n=96) survey participants met the PLUS population criteria.<br><br>93% (n=104) of survey participants were already registered with a GP. Despite this, 65% (n=75) reported that they struggled to access healthcare.	Participants discussed their multiple complex needs, including substance use, homelessness, mental ill health and offending needs.	Patients being observed as being under the influence of substances/ smelling of alcohol.<br><br>Observed patients discussing their multiple complex needs with delivery staff, including obstacles to accessing healthcare, trauma resulting in substance use and/or homelessness, digital exclusion.<br><br>Admin team supported people at the bus sites who met the criteria for the target population but did not access the bus itself. E.g. supporting people with digital access to appointments for their own GP surgeries.
**Effectiveness:**				
Patient satisfaction	n/a	96% (n=108/112) of participants rated their experience of the bus as 'very good' or 'good'.<br><br>95% (n=107/112) felt like they got the help they wanted from the bus.<br><br>99% (n=107/108*) were happy with the wait time.	Patients revealed satisfaction with the delivery of the bus, in particular; the immediate access and 'drop in approach'.<br><br>Patients revealed satisfaction with the longer appointments, so that they could address multiple health issues in one appointment.	Patients informing staff of their positive experience on the bus.

		*n=4 missing data	Patients revealed satisfaction with the delivery staff, finding them to be caring and welcoming, and felt the clinicians listened to them.	
Re-engagement with healthcare	18 clinical referrals were made by clinicians on the bus.	n/a	Patients gave individuals accounts of being supported to register at an appropriate GP practice an/ or re-engaging with their GP's as a result of the positive experience on the bus.<br><br>Staff participants gave examples of patients re-engaging with healthcare.	Admin staff observed supporting patients to register with GP practices.<br><br>Admin staff observed supporting patients to navigate online appointment systems for prescription needs.<br><br>Admin staff observed removing historical 'flags' (virtual markers that alert healthcare staff of potential risky behaviour) from patient records, which previously prevented individuals from accessing primary healthcare.<br><br>Observed patients re-attending the health bus on different days.
Addressing additional health and social needs	n/a	Open comments recorded social history that participants offered as extra information<br><br>Survey captured the additional needs of the population, e.g. social support, supported services they have engaged with, claiming welfare benefits.	Admin staff described how they supported patients with accessing digital records / registering with GPs / other online services.<br><br>Peer workers completing social referrals for patients, e.g. housing or drug and alcohol recovery services.<br><br>Staff revealed that peer workers offered wound-	Observed peer workers making social referrals into housing or drug and alcohol recovery services.<br><br>Peer workers observed offering emotional support to patients, offering transport to/from bus appointments or to hospital appointments, and attending clinical appointments with some patients for extra support.<br><br>Harm-reduction approaches tailored to PWUS observed on site, e.g. offering wound-care packs, testing for BBV.<br><br>Host locations offering support to address additional needs e.g. free food, hygiene facilities, social connections, recovery support, and welfare support.

			care packs and BBV testing to patients.<br><br>Host locations offering support to address additional needs e.g. free food, hygiene facilities, social connections, recovery support.	
Capacity building amongst services	n/a	n/a	Primary care staff expressed improved confidence and skills of working with PWUS because of their involvement with the pilot.<br><br>Staff revealed that the intervention has facilitated ongoing relationships between community services and drug and alcohol recovery services.	Peer workers delivered Naloxone training to research team and primary care staff.<br><br>Peer workers delivered 'Recovery ally training' to primary care staff.<br><br>Observed primary care staff becoming more confident working with PWUS, as the pilot went on.
**Adoption:**				
Organisations willingness and ability to be involved	n/a	n/a	Staff revealed the eligibility of organisations being involved in the pilot, e.g. infrastructure requirements for the bus to be delivered.<br><br>Staff revealed the high need for healthcare support for PWUS, and	Observed ongoing discussions from the delivery team regarding wider partners involvement in the pilot, the barriers/facilitators of this.

			therefore organisations were keen to be involved.	
Value of existing relations and infrastructure	n/a	n/a	Staff participants revealed that pre-existing relationships between peer workers helped them to become involved in the pilot.	Observed ongoing discussions from the delivery team regarding wider partners involvement in the pilot, the barriers/facilitators of this.<br><br>Observed pre-existing relationships between peer workers and host location sites.
How the bus was integrated into the wider care networks	n/a	28% (n=31) heard about the bus through word-of-mouth by a support/community worker.<br><br>28% (n=32) found the bus opportunistically.<br><br>18% (n=20) heard about the bus through word-of-mouth by a friend.<br><br>12% (n=14) found the bus through 'other' means.<br><br>7% (n=8) heard about the bus from a poster displayed in a service.<br><br>4% (n=4) heard about the bus from other healthcare providers.	Staff participants revealed that posters were distributed from their organisation to reach target population.<br><br>Weekly mailing list to wider partners to update on bus location / information.<br><br>Patient interviews revealed a dominance of word-of-mouth promotion.	Witnessed posters at community venue sites.<br><br>Observed who attended weekly catch -up meetings and the impact this had on their dissemination of bus information to potential patients.<br><br>Witnessed peer workers ringing up / or attending different community services (e.g. supported accommodation, or homeless drop-in services) to promote the bus.<br><br>Weekly mailing list to wider partners to update on bus location / information, which grew in numbers throughout the pilot.

		3% (n=3) heard about the bus from a poster advertised online or on social media.		
**Implementation:**				
Clarity and expectations of bus services and staff roles	n/a	n/a	Some staff revealed uncertainty of the bus's aims, target population, and services offered.<br><br>Some staff revealed uncertainty of the roles and responsibilities of the bus delivery team.	Observed differences in clarity of the bus's aims, target population, and services offered based on the staff member (or their organisations) involvement in the pilot. E.g whether they attended weekly catch-up meetings or how often they hosted the bus at their site.
Staff qualities and knowledge	n/a		Staff revealed that ample time and consideration was given during the recruitment process for the primary care team, ensuring that the staff had the necessary skills to support the target population.<br><br>Staff gave examples of when they had utilised their pre-existing networks within primary care to refer patients into secondary healthcare services or to	Observed the admin team as being able to organise appointments effectively and flexibility – at times appointments needed to be re-organised based on priority of patient need. Admin team were skilled at communicating this to patients.<br><br>Observed the admin team liaising with external admin workers in primary care to try and register a patient who did not have a mobile phone or fixed address so that they could be referred to hospital. They were able to forward the referral details to the host location address that the bus was visiting.<br><br>Peer workers delivered Naloxone training to research team and primary care staff.<br><br>Peer workers delivered 'Recovery ally training' to primary care staff.

			be registered with a GP practice.<br><br>Staff from wider partner sites described the delivery team as fitting in well with their own services / their services values.<br><br>Patient interviews revealed that the full delivery team (clinical, admin, bus marshal, and peer workers) were perceived as being good at their jobs, being friendly and welcoming and providing a good service.	Harm-reduction approaches tailored to PWUS observed on site, e.g. offering wound-care packs, testing for BBV.
Access to electronic medical records / information systems	n/a	n/a	Staff revealed the importance of having electronic medical records / information systems, to access patients GP records, leave clinical notes, and facilitate onward referrals.	Observed the role of having electronic medial records / information systems, e.g. registering patients onto the bus, accessing patient records, and facilitating onward referrals / tasks.
Flexibility of service	n/a	n/a	Staff appreciated the flexibility of the operational team and how the service could be adapted quickly to suit the presenting needs of the population, e.g. when uptake was low they would try a new location.	Weekly meetings to reflect on the intervention and meeting the needs of the population. Could respond and adapt quickly to suggestions, e.g. changing the location or changing the delivery times to match an existing service at host location.

Consistency	n/a	n/a	Staff revealed the benefits of consistency for building trust and familiarity with PWUS population.<br><br>Staff revealed the benefits of consistency of location for advertising the service to PWUS, e.g. knowing the bus was always at the same venue on one day so could always promote to their service users.<br><br>Patients who used the bus at the consistent location towards the end of the pilot revealed that they had been informed of the bus through their friends who had attended previously.	Observed that throughout the pilot, a pattern was established where one session was kept at a consistent location where uptake was high.<br><br>While at host sites, some staff felt that it was easier to promote the service to PWUS when the venue was consistent, e.g. weekly or fortnightly at the same venue.<br><br>Observed admin staff building rapports with the PLUS population at the consistent location/day, becoming familiar with the population on a first-name basis.<br><br>Observed the admin team liaising with external admin workers in primary care to try and register a patient who did not have a mobile phone or fixed address so that they could be referred to hospital. They were able to forward the referral details to the host location address that the bus was visiting. The consistency of the location meant that details of the referral could be passed on to the patient.
Locations	n/a	n/a	Patients revealed how they already attended the host locations, which helped them to feel comfortable in the setting.<br><br>Patients revealed that they would have a free meal or take part in an activity while they waited for their appointment.<br><br>Staff emphasised the importance of meeting	Observed differences in bus uptake depending on the location visited and how well attended this was by PWUS.<br><br>Observed patients having a free meal, taking part in an activity or an appointment with recovery workers, while they waited for their appointment.

			PWUS at a comfortable / familiar location, so they are more open to engage with healthcare support.	
**Maintenance:**				
Funding	n/a	n/a	Staff participants acknowledged funding as the main barrier for continuing the pilot.	Observed discussions from delivery team around on-going funding streams and how this impacts the pilot.
Long-term capacity and resources	n/a	n/a	Staff participants revealed the challenges with long-term capacity, e.g. e.g strain on their resources, particularly their time involved with the intervention.	Weekly catch-up meeting attendance rate dropped towards the end of the 6-months.<br><br>Observed that some host locations provided more of their own resources than others, therefore their ability to engage in long-term initiatives will vary.
