# Supplementary material for: Findings from the Process Evaluation of a Mobile Health Clinic Designed to Improve Equity of Access to Primary Healthcare for People with Substance Use Disorders and/or Homelessness in One Region in the North East of England, UK
Source: Healthcare (Basel). 2026 Mar 6;14(5):670. doi: 10.3390/healthcare14050670 (PMC12985337; doi:10.3390/healthcare14050670)
Supplement: Supplementary file 1 [file healthcare-14-00670-s001.zip › healthcare-4125533-supplementary/Supplementary S8 - Additional Interview data.pdf]

Additional Qualitative interview quotes

	**Qualitative interviews**
**Reach:**	'Just obviously going down the [Church] for breakfast on a Friday, I thought, obviously, while it's there, I might as well give it a quick-' … "I was homeless, so obviously going down there for breakfast on a Friday, and that being there, that's ideal' (Participant 5)<br><br>'Yeah, sometimes, because I'd been drinking a lot, I had, like, a couple of seizures (Participant 7)<br><br>'because we were chatting about that I was a recovering alcoholic and stuff like that, so yeah, she [clinician] was lovely' (Participant 8)
**Effectiveness:**	
Patient satisfaction	"I felt more validated because I could talk. Even talking through all of [my health issues] was such a novelty to be able to say that to a doctor. " (Participant 2)<br><br>'Yeah, they were all friendly, welcoming, all that, like. Obviously when you go to the doctor's, you feel like you've got to be kind of in and out, or they try and, like, get out of the appointment by just giving you a prescription, kind of thing, whereas obviously ___ time, haven't you know, like, they didn't rush you' (Participant 5)<br><br>Because this, you know, is accessible, easier, you know, you don't have to be on queue for anything, you don't have to this, you know, it's very relaxing. You know, they are very good. They like community, they like, you know, family. Things like that. So. Just feel relaxed and comfortable with them. Yeah. And also seeing the professional job they're doing to others, you know, So I had confidence, yeah. In them. (Participant 9)<br><br>'Well, their [admin team] whole manner, their attitude. They're really polite. Even if I… They'd see where I was sat when I registered. If I wasn't there, they'd look around, they'd come and find me. In a doctor's surgery, if they're shouting your name and you don't respond, that's it, you've missed your [appointment]- here, they'll come and find me (Participant 14)<br><br>'So I also think the bus is good for promoting positives about health professionals. So we're talking about a community that doesn't have a lot of positives to say about health people' … 'So they're complainers, they moan about the doctors, they moan about the GP, they moan about the receptionists, they moan about the lack of appointments, they moan about the amount of time, they moan about how their prescriptions are not right, they're a very negative community, but actually, they came off that bus really positive' (Staff participant 16)

Re-engagement with healthcare	“And she was very helpful, the woman [Clinician]. Like I say, she got me a doctor- She got me back at the surgery I was meant to be at, so I'm grateful for that'.(Participant 4)<br><br>'I think that worked really well. The fact that the one's that did come, from my experience, must have found it really beneficial because they got on a second and third time if they weren't getting where they needed to be. So they've obviously found it approachable, a good service ... They don't feel unwelcome. They've got on and then got back on again, that was a good sign' (Staff participant 5)<br><br>'But more so, for me with my clients, it provides, like, a bridge. So the ones who I couldn't get registered with a GP and it was constantly like, “You need to see your GP,” you build something so up in your mind that you become really anxious about it. So if they go and see somebody on the bus, they have that first point of contact with a healthcare professional. They find out that they don't get judged, that they feel better, and then I saw people actually going to register with a GP and get checked.... I think that's the biggest value of it... [Researcher 1: Okay. That's amazing]... that now they've got a practice and they're getting sometimes repeat prescriptions for the first time. So that's the biggest benefit that I saw to it' (Staff participant 6)<br><br>'Yeah, yeah, yeah. I would say, I remember one young lad. Well, initially he'd been trying to register with a GP, but because he didn't have a mobile phone he was finding it really difficult, because often it's all done online now, isn't it?' ...' They wanted him to take a photograph of himself and all that. I think in that case one of the support workers was going to go down to a surgery with them and help them do all the paperwork manually' (Staff participant 18)
Addressing additional health and social needs	They were perfect. Because, as I say, they picked me up, dropped me off (Participant 11)<br><br>So immediately seeing them, even before I took the medication, I already started getting better because of the treatment I received. They were like mothers and fathers, you know, so pampering, so h.. helping emotionally... You know, they were like counsellors as well as well as you know, like that. So I was really impressed (Participant 9)<br><br>'Yeah, because [staff] says it's quiet here on a morning because everybody goes then. It's just good. Everything's together. They go there, have their brekkie and fingers crossed, they go and see the GP and then they'll come here to get a shower, get their clothes cleaned. I'll encourage that, “Get

	yourself there, do that and then go and see [homelessness support service] after." (Staff participant 9)<br><br>'I think it was really useful because say, for example, things like that lad not having a phone or them maybe just arriving in the area and not knowing where to go, we wouldn't have really known, but the support workers obviously dealt with that type of thing all the time and they were quite good. They could help him to get a bed in a hostel for the night and things' (Staff participant 18)<br><br>But then what we've also found is in, kind of, the other groups that I've had at Drug and Alcohol Service 3, those who have accessed the health bus, and have been to [Church], have found kind of a community, because it's like, "I can go there, I can get fed, and get seen, I'll meet someone I know, at least, so I've got a social connection," it's not entirely kind of recovery focused, it is purely social, and they're getting their health needs met (Staff participant 13)
Capacity building amongst primary care.	'I think the shock factor of some of the sites or some of the you know like you know when somebody comes in and they're highly intoxicated, the misbehaving or you know I think at first it might have been a little bit of a shock to the system a little bit. Oh, wow. I don't know this is oh, look at him... And then that changed just like it was normalised and there was care and compassion. And so I think even the admin. I think they went through a process, you know, that things were really different to what that we used to work. And you know the the environment was different. The people were different. It wasn't a typical sort of process that they were used to' ... ' But I just think as it moved on, that completely changed. Yeah, I think that changed. And I think it's like you're saying all of the staff members and that sort of especially the admin staff and you know on some of the [organisation 1] workers have sort of their attitudes changed' (Staff participant 2)<br><br>'And to be honest, at the beginning I needed the support workers around to guide us as to *inaudible*... So I think I came in it with not a lot of experience of the plus population and that's changed in the last 6-7 months now we've been doing it now I've got much better insight into it' (Staff participant 4)<br><br>'I interacted with a couple of peer recovery workers and that stimulated an ongoing relationship with [drug and alcohol service 2] which has been really positive'... ' Basically, the individual who leads the [drug and alcohol service 4] session in [Place name 11], they were looking for a venue so they now meet here on a Monday. So, yeah, it's been really fruitful. I think that, kind of, was formed through the health bus relationship so, yeah, I'm quite grateful for the bus for that reason really' (Staff participant 15)
**Adoption:**	
Organisations willingness and ability to be involved	'you know, like we had the [location]. I know that the need in the [location] is massive. Everybody knows that the needs massive' (Staff participant 2)

	‘There are lots of Churches doing what Churches do, feed people, but there’s nobody doing the wraparound support, the drug and alcohol support. There is in isolation, they are doing it, but to have a hub where people can come and do everything… Food is a big part of that, healthcare is a big part of that, so we obviously help people to get appointments but then they struggle . Having something that you can just say, “It’s there on Friday between 8:00 and 12:00, go up and be seen,” (Staff participant 9)
Value of existing relations and infrastructure	‘We were basically enhanced access pilot and another service. So we already had the buy in of the practices that didn't have to do anything additional for that’ (Staff participant 4)<br><br>‘Yeah. I mean, obviously, she would ring up, occasionally, just to flag things up about the bus and what have you but, I mean, I’ve known [Peer worker 1] for years. She just knows we exist so she would send stuff to us and then, if there was anything different, she would let us know. Obviously, she was very much involved. I actually knew the nurse on the bus as well, [nurse 4] from our Church, a retired nurse. She said, the other day, “I told you’d I’d end up in your line of work eventually. I’m not working for you but I’m doing it with the homeless, which is great.” So, obviously, there was a connection there straight away so that was good’ (Staff participant 9)
How the bus was integrated into the wider care networks	‘So we have residents’ meetings here, it would’ve been mentioned in the residents’ meeting, of the new service, where you could access it. Also we have noticeboards like these in our hostel. And we would normally stick the noticeboard on..’ (Staff participant 14)
**Implementation:**	
Clarity and expectations of bus services and staff roles.	‘Because I think I get a little bit confused about whose responsibility it is and I think that would be better just to clear up and get more on board. Like we'll have somebody, if you can't be there on site, we'll have a phone number that everybody can ring to find out about housing for somebody’ (SPS004)<br><br>‘I think it was limited in its skills. So we were told that they would take bloods, they would do this, they would do that, and none of that happened’ (Staff participant 16)
Staff qualities and knowledge	“Only with one patient. He needed to be seen urgently, but he wasn’t registered with a practice, so I registered him with one of ours, and then I liaised with our secretaries. Because he didn’t have a phone number that the hospital would be able to contact him to follow up, and addresses, and all of this for him to get the appointment. So, I liaised with my secretaries. They got onto the hospital, got him a date and everything while he was with us, and he was seen” (Staff participant 10)

	'It seemed, immediately, like they got the point. They were up for it. I thought it was quite telling, as well, that, even though there wasn't a good take-up, there wasn't a lot of frustration. It was kind of like, "Well this is the nature of the beast," almost. They seemed quite open to the fact it might take a bit of time, it might be slow going at first. I don't know whether the people were deliberately selected because they were, perhaps, a little bit more compassionate or a little bit more patient but they certainly seemed that way' (SPS015)<br><br>So for me it was getting somebody with the right skill set with the right sort of ethos. Do you know what I mean? That kind of thing and and wanting to to do this kind of work because I wanted patients to go away with a positive. Attitude and a positive experience of the service. So we maybe did a little bit more screening of of who we allowed to work on the bus as well, so. We're always challenged with staffing, but I don't, there was maybe a little bit more this time just because we wanted to get the right people' (Staff participant 19)
Flexibility of service	'The drop-in element. And the timing. I think the timing is really critical. If a bus is going to work it's got to come when the community is there. Especially a broken community, because they've got no structure to their lives, and they're not good at keeping appointments. So that's where it has worked really well for us. You've made the appointments by being here. So that's been amazing' (Staff participant 11)
Consistency	'yeah, it's good for me to know, "Just get yourself down there," do you know what I mean, especially on a Friday morning when you find people- like, say I do the Friday one, "Get yourself up there, get yourself a breakfast, go and see the doctor and then come in here and get a shower," and that. It's just good everything's all together' (Staff participant 8)<br><br>'Yeah, it was too erratic. So, for me, it was people would come in and ask for it, but it wouldn't be here. So [Place name 5] people need to get into the mindset of, "That bus is there every-" but we couldn't promote it, I felt like it wasn't fair to promote it as being here every Wednesday, because it wasn't. So I don't think the trial period was long enough' (Staff participant 16)
Locations	'Well, everybody likes that church, that's the only reason I keep coming back, and it is comfortable, I mean, it's ... location, and... I mean, [staff member] that's in there is an amazing bloke. So, basically, I think everybody's comfortable' (Participant 6)<br><br>' [church] was good, because all the people that were in there waiting to get on the bus, or had already been on the bus, were all sat in a group and chatting, having a coffee and stuff. Yeah, it was good. But, obviously, you've got the same here as well' (Participant 1)<br><br>'I think we're talking about people who've been marginalised by society for years and years, who are used to stares and used to not feeling like they exist as they're going into normal services. Then you're catching them at a place where they generally feel safe and they've got a sense of community and

	they’re around people who are in the same sort of boat. They feel much more comfortable to access things’ (Staff participant 5)<br><br>‘Because they weren’t going to a doctor’s surgery or a place by themselves. They had people here that they trust, that they know, they’ve got that support in place and it wasn’t like a big thing where I’ve got to make an appointment and I’ve got to go to an environment I might not feel safe in. The way that you had it set up was, “Right, it’s your turn then, it’s your turn,” so they could see other people in their peer group doing the same thing. So they had so much support in place. They didn’t have to sit in a waiting room. They could sit here and play the bingo and do what they do every week. So that’s the biggest thing, the biggest benefit to it’ (Staff participant 6)
**Maintenance:**	
Funding	‘Trying to keep it going, that was difficult. So the funding was a difficult thing to be able to say and keep people engaged . There's lots of services that the sessional staff can pick up. Now we're going in the winter, they've got winter pressure hubs to get funding, so they're going to be pulled’ (Staff participant 4)<br><br>‘Well the barrier is just funding, isn't it? I mean that’s going to be the only reason it doesn’t continue’ (Staff participant 9)<br><br>‘The funding element has to be there and the commitment to that funding element because it has to be something that's consistent. You know, we can't people, people know it's going to be there, whether it's filled that week or not filled that week etcetera. So it's managing those expectations, going to different areas, advertising it’ (Staff participant 19)
Long-term capacity and resources	‘And, as a community, in [location] and [location], which is quite high deprivation, they’re very used to flash-in-the-pan schemes, that start up with funding, and then disappear’ (Staff participant 16)<br><br>‘I think part of this is absolutely because of the way that I was drawn into the conversation. It was very much… Initially, it was just they were looking for somewhere to site the bus and then it was, “Actually, we need to plug it in. It would be really good if we could have some indoor space. Actually, can we have that space as well?” So it, kind of… I guess it evolved from just using the carpark to, kind of, taking over a large chunk of the building, which I was absolutely fine with. Very happy with that, I just felt I was probably committed before I realised what I was committed to, if that makes sense?’ (Staff participant 15)
