# Supplementary material for: Findings from the Process Evaluation of a Mobile Health Clinic Designed to Improve Equity of Access to Primary Healthcare for People with Substance Use Disorders and/or Homelessness in One Region in the North East of England, UK
Source: Healthcare (Basel). 2026 Mar 6;14(5):670. doi: 10.3390/healthcare14050670 (PMC12985337; doi:10.3390/healthcare14050670)
Supplement: Supplementary file 1 [file healthcare-14-00670-s001.zip › healthcare-4125533-supplementary/Supplementary S9 - Additional observational fieldnotes.pdf]

Additional observational fieldwork extracts

	Observational fieldwork notes
**Reach:**	Just before the bus was about to close, a man came into [venue] asking to access the bus. As soon as he entered [the peer worker] was there to greet and make him feel comfortable providing lots of reassurance. He said he felt uncomfortable being in the [venue] as he had previously been 'thrown out'. As he sat down, I could smell alcohol on him and he seemed agitated. He described using alcohol and drugs for many years to mask lifelong trauma and mental ill health, and how difficult it was to stay well so he could be there for his family. He described previously accessing [primary care mental health support] for this trauma and although initially positive, once his worker told him they were changing roles and handing him to another colleague, he felt the rapport and trust had been lost and he didn't continue. He said difficulties applying for access, or waiting times for assessments (for his undiagnosed PTSD) massively put him off seeking support or healthcare. Although he was registered with a GP he had not been for years. (Observations, month 2)
**Effectiveness:**	
Patient satisfaction	She sat and waited with someone and got a hot drink whilst she waited and seemed happy whenever I updated her on her wait (Observations, month 2)<br><br>Another lady who visited the bus took part in the survey. She described her main reason for visiting the bus was to check her blood pressure, however had spent longer speaking with the GP about the emotional support she and her daughter were able to access relating to her daughter's mental ill health, as this lady cares (informally – not collecting any benefits) for her daughter. She described how much she appreciated having time and how supportive the bus GP had been and that she had had negative experiences with her own GP so although registered wouldn't go back to them for support. (Observations, month 2)<br><br>[Peer worker] had encouraged a gentlemen that had not seen his GP in a number of years to go on the bus, and he was happy to wait on the sofas until [admin] was available to check him in (Observations, month 3)<br><br>They felt the doctor on the bus was very thorough and were really appreciative of being seen. He really appreciated the peer support and shared that he wanted someone who could relate to his experience because they had been in a similar position before (Observations, month 4)<br><br>One patient has been in regularly over the past few weeks and told the team that he was disappointed that the service was ending as he was really helped by it, as he has been dismissed by other health professionals and going to the bus was the first time someone had listened and agreed that he should be concerned (Observations, month 6)

Re-engagement with healthcare	5 patients seen on bus (one visiting for second time) (Observations, month 3)<br><br>Busy day at the bus approximately 9 patients seen (at least 1 repeat attender (Observations, month 4)<br><br>A patient came across today and said “Are you the counsellor?” and [bus service manager] said ‘This is [peer worker name]”. The patient then spoke to [peer worker], who then spoke to [bus service manager] who tried to deal with the problem which was that the patient wasn’t able to get a prescription for their anxiety and depression medication. [Bus service manager] was later helping the patient to navigate the online patient appointment system as the patient doesn’t have a phone. (Observations, month 5)
Addressing additional health and social needs	[Peer worker] shared that due to his conversation with that individual, they had shared with [peer worker] that they were struggling with their use of substances/alcohol and wanted support. [peer worker] had arranged to support them to attend a [drug and alcohol service] support group later that week (Observations, month 1)<br><br>[Peer worker] was there for a little while. I asked whether either of the two gentlemen he had met last week at [Venue] came to his support group. He said one of them did, and that he felt even the one who didn’t attend was now aware of the group, so he felt this was positive (Observation, month 1)<br><br>There is clearly a strong support network at [Venue]. A lady came in crying and was sat with [Staff member] to talk through her situation. Another lady who visited the bus had a small child with her. One of the volunteers in [venue] looked after the child while the lady was seen to (Observations, month 1)<br><br>One gentleman came in and [bus delivery staff] shared that he had accessed the bus another week. He asked to visit the bus again due to his mental health as he was feeling low and wanted to speak with someone. The man talked with [peer worker] for some time. The gentleman left and returned shortly after with papers he wanted to show [bus delivery staff]. Through discussion with [bus delivery staff] it was discovered that he had been given an appointment through Talking Therapies, however due to an administrative error on the letter, it stated that he had missed his appointment. English was not his first language, so [bus delivery staff] took some time to understand and try to explain to the gentleman what had happened as he had been confused. [bus delivery staff] was able to sit with this gentleman and called Talking Therapies on his behalf. She was able to reorganize the telephone appointment for this patient and also update his mobile number which had changed (meaning they would not have been able to contact him prior to this). The man did visit the bus again to see [doctor]. I also heard [peer worker] making calls to a [drug and alcohol service] outreach worker to set up an assessment for this gentleman for the following week (Observations, month 2)

	Once he had been seen by the GP he had something to eat from the [Venue] staff. He talked about needing clothes and said he wanted to reengage with recovery services because he wanted to make a change. He said he usually sees [staff] at the [venue] who has been looking after him recently. He completed a survey and shared that he is drinking alcohol and has mental ill health. [peer worker] talked with him again and said he would meet him and go along to a group support session with him, so he didn't need to do it alone. [peer worker] shared some of his own recovery journey and provided lots of reassurance. When he was leaving, the man said he felt like this was the first time in ages that he felt that he had been listened to and that he appreciated everyone being so warm and welcoming. He called [peer worker] a 'legend' (Observations, month 2)<br><br>[Bus delivery staff] was supporting the gentleman from last week with the shakes for his diabetes – due to his digital exclusion. After speaking with someone from the company last week [Bus delivery staff] had arranged for the link to the service to be sent a different way after they had set up an email account for the gentlemen, however they were having issues as the password (either for the email or the link to access the service?) was not working so [Bus delivery staff] was supporting him with this. I think [Bus delivery staff] mentioned something about him getting access to a better phone, not sure who this was through (Observations, month 3)<br><br>After attending they spoke with [Venue] staff and identified that they required support with welfare/finances and employment opportunities. They both scheduled an appointment with staff to address these needs (Observations, month 3)<br><br>One PLUS patient at [Venue], wasn't aware of the healthcare bus prior to attending, but used the service and expressed mental health concerns (suicide ideation). Also identified as rough sleeping the previous night, and some risk of exploitation/ safety at his named address. A wrap around approach was delivered to support this patient including staff already based at [Venue] (police and social prescriber) but also staff from [Healthcare provider]and [Drug and alcohol service] support workers (there as part of the health bus programme). Highlighted the benefits of collaborative working playing to different professionals' strengths - for example the [Healthcare provider] could write a letter signed by a doctor to present at A&E to help explain the current situation and signpost to appropriate and quick support, the police were able to provide a phone to the patient so he could be contacted by CRISIS team, the [drug and alcohol service] staff were able to drive to Tesco's to collect a sim card for the patient to use, [Venue] were able to provide the patient with free food. It also highlights the different unmet health and social needs of members of the PLUS population that can be revealed in one meeting (Observations, month 4)

Capacity building amongst primary care.	[Bus delivery team] seem to know a lot of the people at [venue] now so remember their details to get them booked in with an appointment which is a nice touch (Observations, month 6)
**Adoption:**	
Organisations willingness and ability to be involved	Also discussed other venues as [Bus delivery staff] did not seem keen to return to [venue] as it had been so quiet. [Venue] was discussed, and [bus delivery staff] was planning to take the bus there at the end of the session to assess parking space and accessibility for the bus. [peer worker] supports people in there as many are PLUS population and estimates there are around 70 residents who may access the bus (Observations, month 4)
Value of existing relations and infrastructure	[Peer worker] and [peer worker] were there [venue] to provide peer support and clearly already had good relationships with many of the people there (Observations, month 1)<br><br>We talked about alternative venues and all agreed [Venues] could work well. We also discussed [Venue] which seemed another viable option if looking for other locations. [Peer worker] felt there wouldn't be resistance as had initially been expressed with us during interviews last year. He said he knows many people in the community there, so potentially if the bus did visit [Venue] he could visit ahead of time to raise awareness of the bus coming to ensure a better turn out (Observations, month 1)<br><br>[Bus delivery staff] and [Delivery staff] suggested the mental health hub in [Venue]? [bus delivery staff] has a contact there called [name] who [bus delivery staff] is going to contact (Observations, month 4)<br><br>Also discussed other venues as [Bus delivery staff] did not seem keen to return to [venue] as it had been so quiet. [Venue] was discussed, and [bus delivery staff] was planning to take the bus there at the end of the session to assess parking space and accessibility for the bus. [peer worker] supports people in there as many are PLUS population and estimates there are around 70 residents who may access the bus (Observations, month 4)
How the bus was integrated into the wider care networks	The first two patients today came from [hostel] where they said they had seen a poster for the bus and walked down to (venue). (Observation, month 3)<br><br>There were several patients from [hostel] at venue X today. They explained that there is a poster up there with [peer worker's name] on it. It seems like they have been telling each other about the bus. One previous patient had brought his friend who was having a suspected stroke. I think they were prioritised to go on the bus and then advised to go to A&E. [peer worker] took them to A&E. (Observations, month 4)

	Two new patients arrived at about 11.30am who were from [organisation] hub and had been brought by a Peer Mentor. They had all been told about the bus by the [organisation] staff. Another example of partnership working and the word spreading about the bus (Observation, month 4)<br><br>[Staff] asked if she could share on the centre facebook page which [Bus delivery staff] agreed to. [Staff] said this was a good way to reach their community (Observation, month 4)<br><br>Due to initial low turnout, the support workers drove to other locations (Venu, [Venue], [Venue], [Venue], and [Venue] to try and encourage people to come and use the bus / and offer transport there. This approach was successful in bringing one person to come and use the bus, who definitely met the PLUS criteria (Observations, month 4)
**Implementation:**	
Clarity and expectations of bus services and staff roles.	There were sometimes that patients were coming to [research team] to ask about bus related things or for support, rather than the peer support workers. When people are sat having breakfast and there are more people around, it can be a bit hard to point out who is there with the bus. I chatted to [bus delivery staff] who said it would be best if there was always one person from [drug and alcohol service stood outside with the bus who could direct people to where they need to be. It is a bit unclear on whose role it would be to advise the support workers on what to do, as [bus delivery staff] said he didn't feel comfortable directing them, but it is also not the role of the research team (Observation, month 2)<br><br>We discussed how it was important to have two peer support workers there on the day, as [peer worker] wouldn't have been able to help people register [to a GP] whilst also greeting people and encouraging them to seek appointments (Observations, month 4)
Staff qualities and knowledge	[Bus delivery staff] mentioned the value of having [admin staff] who is a very experienced administrator. I observed that [admin staff] has a really lovely compassionate manner with patients (Observations, month 2)<br><br>The initial list was changed as one patient said that they had various appointments to get to so they were moved form 3rd to 2nd in the queue (Observations, month 4)<br><br>The peer workers have information about the patients that we don't always have e.g. 'he's a prolific shoplifter' 'she really looks like she's in a bad way' (Observations, month 6)

Flexibility of service	[Bus delivery staff] had concerns about running the bus here next week because we had seen many regulars to the centre and didn't want to keep seeing the same people (Observations, month 4)<br><br>As soon as we arrived at 8.45am people were approaching the bus to see if they could go on. I think the list was filled by just after 9am, however a couple of people left because they had other appointments. …… [Bus delivery staff] reported plans to change the time of the bus at [Venue] on Friday, going forward. She said they will aim to start at 8am with the bus leaving at 12 noon - last patients will be seen around 11.30am (Observations, month 4)<br><br>The new start time works better as [healthcare provider] are able to get things set up before patients start approaching (Observations, month 6).
Consistency	I briefly spoke to [staff] who runs the [food bank] he offered me a cup of tea and thanked us for being there and said 'as you'll see this is a fragile population' he mentioned people needed to see the bus for a few weeks and to build up trust (Observations, month 2)<br><br>[bus delivery staff] stated most challenging day so far as patients who are homeless with no fixed address and with no phone. They have had to troubleshoot how to get in touch with them to tell them where their appointments are, how can we get in touch with them? They have been giving the address of [Venue] (Observations, month 2)<br><br>Admin staff mentioned how they are becoming familiar with service users and building a somewhat rapport with them. They mentioned a lady who used the health bus at [Venue] was also at [Venue] for breakfast on the Friday. They asked her if she was able to pick up her prescription, as this was the outcome of the health bus service, which she was able to. Highlights that follow up care that can be provided from being in the community. If there had been any obstacles to her picking up her prescription the staff would have assisted her further (Observations, month 5)<br><br>Someone who has used the bus a few times now to get their prescription had brought some friends along to use the bus (Observation, month 5)<br><br>Most patients seem happy to approach bus on their own now (Observations, month 6)
Locations	[Venu] were running their bingo and sausage sandwiches, it was also a sunny day, so it was busy. As before this seemed to help with anyone waiting to be seen on the bus (Observation, month 1)<br><br>As a I came into the [venue] I spoke to a man on reception who said that the bus was very needed and that the people who access the breakfast are in need. He said there had recently been a funeral for a person who accesses the breakfasts. I hadn't heard about this from anyone else. – During his weekly

	talk [staff member] thanked God for helping a women who volunteers at the kitchen with her recovery journey, he also asked us to pray for a member of the volunteers who has throat cancer (or whose family members has throat cancer).We reflected that this is a lovely community spirit and the value of community and support (Observations, month 3)<br><br>Many described social isolation and that they came to the centre to get out of the house, get food, and socialise. One gentleman said despite this he still didn't feel he could talk to people on a deeper level and used humour to mask the difficulties he felt (signposted to [mental health support group] as a local support group) but he also has support in the centre. This seems similar to conversations from others at [Venue] (Observations, month 4)
**Maintenance:**	
Funding	[Staff] mentioned that some funding has been confirmed which might help to continue the bus beyond the three-month period / make it more flexible (Observations, month 1)
Long-term capacity and resources	ANP was questioning why the bus was being parked outside of buildings rather than hiring out rooms in the places (Observations, month 2)<br><br>[Staff member] explained that if the bus was to go to [Venue] next Wednesday, she would contact [Venue], [Venue], [Venue] and go to the [Drug and alcohol service] at [Venue] and [Venue] to tell people about it. They also tell all the people who attend their services about the bus. [Staff member] also mentioned that they have printed a poster that they will put up (Observations, month 4)<br><br>[Bus delivery staff] was concerned that no one from [Drug and alcohol service] had attended the previous weekly catch up meetings and I informed her I had heard [Staff] was off sick at the moment so she contacted other [drug and alcohol service] staff to see if someone else would be available to attend on Thursday to keep them involved (Observations, month 4)
